# A qualitative study of mental health problems among children living in New Delhi slums

**DOI:** 10.1177/13634615231202098

**Published:** 2024-02-23

**Authors:** Prerna Martin, Emily E. Haroz, Catherine Lee, Paul Bolton, Kiran Martin, Rosemary Meza, Elizabeth McCarthy, Shannon Dorsey

**Affiliations:** 1 8783 Department of Psychology, University of California Los Angeles, Los Angeles, CA, USA; 2 Department of International Health, Johns Hopkins Bloomberg School of Public Health, Baltimore, MD, USA; 3 UNICEF New York Child Protection in Humanitarian Action, New York, NY, USA; 4 USAID, Washington, DC, USA; 5 Asha Community Health and Development Society, New Delhi, India; 6 343041 Kaiser Permanente Washington Health Research Institute, Seattle, WA, USA; 7 Department of Psychology, Seattle Pacific University, Seattle, WA, USA; 8 7284 Department of Psychology, University of Washington, Seattle, WA, USA

**Keywords:** children, global mental health, India, needs assessment, qualitative research, urban slums

## Abstract

Children living in urban slums in India are exposed to chronic stressors that increase their risk of developing mental disorders, but they remain a neglected group. Effective mental health interventions are needed; however, it is necessary to understand how mental health symptoms and needs are perceived and prioritized locally to tailor interventions for this population. We used an existing rapid ethnographic assessment approach to identify mental health problems from the perspective of children living in Indian slums, including local descriptions, perceived causes, impact, and coping behavior. Local Hindi-speaking interviewers conducted 77 free-list interviews and 33 key informant interviews with children and adults (N = 107) from two slums in New Delhi. Results identified a range of internalizing and externalizing symptoms consistent with depression, anxiety, and conduct problems in children. Findings included both common cross-cultural experiences and symptoms as well as uniquely described symptoms (e.g., “madness or anger,” “pain in the heart and mind”) not typically included on western standardized measures of psychopathology. Mental health problems appeared to be highly interconnected, with experiences such as harassment and fighting often described as both causes and impacts of mental health symptoms in children. Community perspectives indicated that even in the face of several unmet basic needs, mental health problems were important to the community and counseling interventions were likely to be acceptable. We discuss implications for adapting mental health interventions and assessing their effectiveness to reduce the burden of mental illness among children living in urban slums in India.

## Introduction

India has seen a significant increase in urbanization over the past 20 years, and the country's urban population is expected to have increased from 377 million in 2011 to 600 million by 2030 (Unicef, 2012). This rapid urbanization has been accompanied by a rise in urban poverty from 18.7% to 26.8% in the last four decades (Unicef, 2012). One of the most prominent physical representations of this rise in urban poverty is the growth of urban slums^1^ (Sahasranaman & Bettencourt, 2021). The 2011 Census revealed that over 65 million people lived in urban slums in India, comprising more than 17% of India's urban population (Office of the Registrar General & Census Commissioner, 2011). Urban slums are characterized by high density, lack of water and sanitation, inadequate and unsafe housing, noise and environmental pollution, malnutrition, higher maternal and infant mortality, higher rates of infectious diseases, and limited access to health care and education (Marimuthu et al., 2009, 2016; Sharma, 2015). The quality of life in an urban slum is exacerbated by a lack of legal protection for residents, limited political voice, high crime rates, violence, and discrimination based on sex, ethnicity, caste, or disability, all of which further exclude this group from the rest of the urban population (Jungari et al., 2022; Unicef, 2012).

Children and adolescents in India's urban slums are exposed to many stressors that increase their vulnerability for mental health problems. These include adverse experiences of *deprivation*, such as lack of access to basic services (e.g., water, food, health, education), parental neglect and unstable housing, and experiences of *threat*, including physical and sexual abuse, domestic and community violence, and lack of child protection and safety (Fernandes et al., 2013; McLaughlin et al., 2014; Patel et al., 2007). Multiple U.S. and global studies have documented the increased risk for developing mental health disorders including mood, anxiety, behavioral disorders, and substance use disorders among individuals with adverse childhood experiences of deprivation and threat (Cohen et al., 2001; Duarte et al., 2003; Earls & Carlson, 2001; Kessler et al., 1997; McLaughlin et al., 2012; Patel & Kleinman, 2003; Patel et al., 2007; Wado et al., 2022). In Indian slums specifically, studies have documented the association between physical abuse, sexual harassment, parental mental health disorders, and economic deprivation, and psychiatric disorders among youth (Cheng et al., 2014; Fernandes et al., 2013; Srinath et al., 2005).

Over the past few decades, data from nationwide epidemiological surveys and meta-analyses have estimated a wide range of prevalence rates of psychiatric morbidity among children and adolescents in India, from 1.81% to 35.5%, with higher rates observed in urban areas (Bhola & Kapur, 2003; Hackett et al., 2017; Khairkar et al., 2013; Malhotra & Patra, 2014; Nandi et al., 1975; Pillai et al., 2008; Srinath et al., 2005). However, data from children and adolescents residing in urban slums is more limited. A study conducted in a Mumbai slum found a prevalence rate of 14.8% for common psychiatric illnesses in children aged 5 to 14 years (*N *= 257), which included behavioral disorders (conduct and oppositional defiant disorder), attention-deficit hyperactivity disorder, intellectual disability, and depression (Patil et al., 2013). In another study conducted in an Andhra Pradesh urban slum, researchers found the prevalence of emotional and behavioral problems to be 22.43% among children aged 5 to 10 years (*N *= 370); these rates were higher than those in other urban and rural settings in the country (Bele et al., 2013). A recent study conducted in Lucknow slums noted that approximately 33% of adolescents (aged 12–19 years) suffered from severe mental health problems, and these rates were more likely to be higher among females and younger adolescents (*N *= 590) (Chauhan & Dhar, 2020).

These studies indicate the presence of both internalizing and externalizing problems among children living in Indian slums. However, an over-reliance on Western measures and diagnostic criteria in these studies may limit our understanding of cultural variations in psychological distress among children living in Indian slum communities. To our knowledge, no studies have examined local perspectives on how children and families residing in Indian slums themselves understand and describe mental health problems, what causes mental health problems, and their impact. Additionally, prior research has not investigated *prioritization* of mental health problems in the context of other important basic needs, or local perspectives on how they should be addressed. A large body of cross-cultural qualitative research has shown that variations in culture and context influence the presentation, prioritization, and implications of mental health problems (Betancourt et al., 2009; Bolton et al., 2013; Dorsey et al., 2015; Kohrt et al., 2014; Murray et al., 2006). Obtaining this information from a local perspective is vital to selecting problems that are important to local communities, and to identifying interventions that will be acceptable and feasible for children and families living in slums.

The purpose of this qualitative study was to understand local perceptions and prioritization of the mental health needs of children and adolescents living in Indian slums. This study used a rapid ethnographic assessment method developed for this purpose (Applied Mental Health Research Group (AMHR), 2011) to examine the nature and perceived causes of the most salient psychosocial problems among children living in slums, their impact, the community's current strategies to address these problems, and additional recommendations. To our knowledge, this is the first study to understand these issues among children living in Indian slums from the community's perspective. The goal was to use the study's results to 1) select problems that match community priorities and adapt locally acceptable interventions to target those problems, and 2) design and adapt instruments to obtain relevant assessment tools of intervention effectiveness.

## Method

### Study sites

This study was conducted over a two-week period in August 2016 in two urban slum communities within the capital city of New Delhi: *Ekta Vihar* and *Ambedkar Basti*. The study was implemented as a partnership between the University of Washington and Asha, a nongovernmental organization (NGO) that has been providing community-based health care and education services in New Delhi's urban slums for the past 34 years. *Ekta Vihar* has a population of 3,000 residents, with their religious affiliations being Hindu (95%) and Muslim (5%). Residents’ dominant castes are Rajput (i.e., warrior caste) and several lower castes (e.g., Rajasthani Dhol, Mali, and Valmiki). *Ambedkar Basti* is home to 5,000 residents, with their religious affiliations being Hindu (99%) and Muslim (1%). The majority of residents in *Ambedkar Basti* belong to the Dalit or “Scheduled Caste,” the lowest caste in the traditional Hindu social hierarchy. In these slums, 45% of households have 4–5 members, 22% have 6–8 members, and 5% have more than nine members (Office of the Registrar General & Census Commissioner, 2011). Asha selected these two sites for study participation as they had strong community partnerships and existing infrastructure in these slums that would facilitate a pilot implementation of mental health services for youth in the future.

### Participants

We invited 124 community members to participate in this study, of which 107 provided informed consent (86%). Reasons for declining participation included lack of time due to working multiple jobs or competing responsibilities (71%), participant traveling to their village (12%), and no interest in study participation (17%). Final study participants included 43 children (ages 10–17 years) and 64 adults living in these two slums (*N *= 107) who participated in either or both free-list (FL) and key informant (KI) interviews. FL interviewees included a convenience sample of 40 children (17 males, 23 females) and 37 adults (18 males, 19 females) who had lived in one of the two slums for at least five years and were available and willing to speak with interviewers during the study period. Recruitment was done by Asha study staff who went door to door and visited weekly community groups occurring at the Asha Community Health Center in each slum to share study information. Purposive sampling was used to balance recruitment across age and sex to obtain a range of perspectives on children's mental health problems. Following the FL interviews, in-depth interviews were conducted with 33 KIs (16 males, 17 females) to learn more about potential mental health problems identified in the FL interviews. KIs were identified by FL respondents using a snowball-sampling approach. These were local adult and adolescent residents recommended by the FL respondents and other slum community members as being particularly knowledgeable about the psychosocial problems of children living in those slums. In our sample, KIs included community health workers, local leaders in the slum (e.g., slum president, women's group leader), and members of women’s and youth groups who voluntarily carry out other program activities at Asha. Priority was given to individuals mentioned by multiple FL respondents. Professional health care providers (e.g., social workers, nurses, physicians) were excluded due to the possibility that these individuals may report on problems from the perspective of their training rather than the community's experience.

### Procedures

We used a rapid ethnographic assessment method based on a grounded theory approach developed by Johns Hopkins University's Applied Mental Health Research (AMHR) group to understand local representations of psychosocial problems of children living in slums (AMHR, 2011, 2013). This qualitative assessment is the first of eight modules within their Design Implementation Monitoring and Evaluation (DIME) framework that has been used in multiple low- and middle-income countries (LMICs) to assess locally reported mental health needs in a community (e.g., Betancourt et al., 2009; Bolton et al., 2012, 2013; Dorsey et al., 2015; Murray et al., 2006). The study procedures were approved by the Institutional Review Board at the University of Washington. Interviewers read out IRB-approved recruitment scripts, consent forms, and assent forms to participants, and all participants provided verbal informed consent. All staff were trained in research ethics. At every stage, precautions were taken to protect participant identities and minimize adverse effects that might result from the interviews.

Sixteen Asha staff who had worked extensively in New Delhi slums were selected as interviewers to build local capacity and increase interviewer acceptability in the community (11 females, five males). All interviewers had a bachelor's degree (six in social work, one in nursing, nine in other fields) and were employed at Asha as team leaders overseeing Asha's health and education community intervention across various slums. Of the 16 interviewers, five had prior data collection experience and two senior staff members with the most experience were selected as supervisors to oversee recruitment, data collection, and analyses. Study staff were fluent in the local language Hindi. Interviewers received two days of training in research ethics, qualitative interviewing methods, and data analysis by the first author (PM). The training involved didactic and experiential learning via role plays with feedback, emphasizing open-ended, non-leading questions and probes to reduce interviewer influence and bias. The first author (PM) observed each interviewer's role plays prior to starting data collection to ensure readiness in qualitative methods. An additional day of training focused on KI interview procedures was provided immediately before starting KI interviews. During the trainings, interviewers also translated the FL and KI interview questions into Hindi to ensure the most appropriate translations for the target population.

Interviewers worked in pairs, where one interviewer asked questions and the second recorded responses verbatim by hand. The recorder could interject when he/she identified a problem in the interview (e.g., a leading question or insufficient probing). All interviews were conducted and recorded in Hindi. At the end of each interview, the interviewer and recorder combined notes into a single record and both agreed on the accuracy of the final interview text before proceeding. Supervisors reviewed all interviews at the end of each day and provided feedback about where additional probing may be needed.

### Free-listing interviews

FL interviewing was used to generate a priority list of perceived problems of children living in slums from the perspective of local community members. During the interview, all FL participants were asked the following open-ended question: “What are the problems of children living in slums?” Participants provided a list of problems, along with brief descriptions of each problem. Interviewers were trained to identify potential mental health problems, defined as any problem related to thinking, feeling, or relationships (AMHR, 2011). For each potential mental health problem, interviewers asked participants to identify community members who were knowledgeable about that problem, and to whom people would go when a child had that identified problem. These names were recorded for later contact as potential KIs. Each FL interview took approximately 60 min. Interviews continued for three days until study staff determined that no new information was being obtained.

### Key informant interviews

At the end of the FL interviews, we held a meeting with our primary stakeholders, i.e., Asha leaders, Asha staff that may be involved in mental health programming in the future, and study staff, who collaboratively reviewed FL interview analyses and selected three potential mental health-related problems among children to explore further with KI interviews: (1) *Chedkani* (harassment), (2) *Ladaee* (fighting), and (3) *Depression*. Per DIME methodology, these problems were selected by stakeholders based on multiple criteria, including frequent mention by respondents, potential severity of impact, not currently being well understood or needing further exploration to plan for interventions, and those most likely to be addressed by Asha (AMHR, 2011). While fighting and harassment were not specific mental health problems, our stakeholders determined that they should be explored due to their relative importance and frequent mention of having a clear mental health impact on children in the FL interviews. While depression was not as frequently mentioned in the FLs, our stakeholders decided to explore this further as it was not well understood in this population. A fourth problem (*nasha*; substance use) also met many of the above criteria but was not selected for further probing based on Asha's decision to address other mental health problems before substance use, due to capacity and available resources.

The purpose of the KI interviews was to get in-depth information about each selected mental health-related problem. All KIs were asked to report on the following questions for each of the three selected problems (using harassment as an example below):

1. *Nature of the problem (signs, symptoms, etc.)*: What happens when children are harassed? What are the different types of harassment / what does this problem look like in children?
2. *Perceived causes*: Why are children harassed?
3. *Impact*: What is the impact of harassment on children? How do they think, feel, and behave when they are harassed? What is the impact of child harassment on the family and the community?
4. *Current response*: What do people currently do about the problem of child harassment?
5. *Recommendations*: What should be done about the problem of child harassment?

KI interviews took place over a five-day period, during which all KIs were interviewed at least twice, with each interview being 60–90 min in duration. Repeat interviews allowed interviewers to gather information that may have been missed the first time or to obtain more details after having established increased trust and comfort. Interviewers probed on each of the questions until KIs had no new information to share. Per DIME methods, both FL and KI interviewees were asked to respond based on their general interactions with children in slums rather than about themselves or their own children to maintain privacy, increase reporting of stigmatized or sensitive problems, and report widely held knowledge rather than personal opinions (AMHR, 2011).

### Data analysis

Study supervisors and interviewers conducted FL and KI data analyses in Hindi using domain analysis techniques. This involved identifying *cover terms* that described the overall problem, followed by *included terms*, which were other ways of describing that problem or various concepts captured by the cover term. For FL analyses, data from all interviews was reviewed to create consolidated lists of adult-reported and child-reported problems, and frequencies of how many participants endorsed each problem. Decisions about whether two differently worded items referred to the same or different problems were made by group consensus. If study staff agreed that two items referred to the same problem (e.g., “not going to school” vs. “not attending school”), staff selected the wording that was most likely to be understood by the community. When disagreements occurred, items were listed as separate problems. These composite problem lists were then organized by frequency of unique participants endorsing that problem. Per DIME methodology, this relative frequency was used as an indicator of problem prioritization, where more frequently mentioned items were considered to be relatively more important to the community (e.g., Bolton et al., 2013; Murray et al., 2006). The adult-reported and child-reported composite FLs were translated to English after analysis by the interviewers and first author.

KI data were analyzed following a similar procedure. Study staff were divided into three teams, one for each of the three KI problems. Each team reviewed all the KI data for the specific problem that was assigned to them, creating lists for the nature of the problem (signs and symptoms), the perceived causes, the impact of the problem, what people currently do about the problem, and what people should do about the problem.

## Results

### Free-listing results

*Problems Identified by Adults.* Twenty-seven problems were identified by three or more adult participants (Table 1). The most commonly endorsed problems were lack of basic needs (e.g., shortage of clean water and functioning toilets), alcohol and drug use (e.g., tobacco, marijuana, opium), and school-related problems (e.g., missing school, not studying). Study staff identified 14 of the 27 problems as potential mental health-related issues based on our specified criteria. Fighting and harassment were two mental health-related issues that were the fifth and sixth most commonly reported problems, each reported by over two-thirds of the sample. Other child experiences in the slum that either indicated a mental health problem or could *lead* to mental health problems included verbally aggressive behavior by children (e.g., abusing, swearing), children engaging in “bad behaviors” (e.g., joining gangs), discrimination (e.g., due to sex, caste, or religion), gambling, child neglect (e.g., parents leaving children unattended), inappropriate sexual relationships (e.g., young children having consensual sex), disrespecting elders, stealing, parents beating children, depression, and child prostitution.

**Table 1.** Problems of children identified from free-list interviews with adults (n = 37)^a^.

Problem	Frequency (%)
Lack of basic needs (e.g., clean water, toilet facilities, electricity, clothing, small & unstable homes)	37 (100)
*Alcohol & drug use	34 (92)
School-related problems (e.g., children missing school, can’t study)	31 (84)
Squalor/bad sanitation	29 (78)
*^+^Fighting	25 (68)
*^+^Harassment (verbal, physical, and sexual harassment)	25 (68)
Bad environment (e.g., excessive noise, parents fighting)	16 (43)
Child labor	14 (38)
*Abusing/swearing (verbal aggression)	13 (35)
*Bad behaviors (e.g., falling into bad company, spending money for school fees on other things)	12 (32)
*Discrimination (e.g., gender based; girls not allowed to study)	11 (30)
Child marriage	10 (27)
Physical health problems/illness	9 (24)
*Gambling	8 (22)
Lack of child rights (e.g., not treated when sick, not sent to school)	8 (22)
Poverty	7 (19)
*Child neglect	5 (14)
*Inappropriate sexual relationships	4 (11)
*Disrespecting elders	4 (11)
*Stealing	4 (11)
*Beating children	4 (11)
*^+^Depression	4 (11)
Unemployment (adults)	4 (11)
*Child prostitution	4 (11)
Narrow-mindedness (negative thinking, bad thoughts)	4 (11)
Lack of awareness (parents)	3 (8)
Jealousy	3 (8)

*Notes*. ^a^Problems mentioned by three or more respondents. *Potential mental health problems or problems that may have a mental health impact. ^+^Problems that were selected for in-depth key informant interviews.

*Problems Identified by Children.* Twenty-five problems were identified by three or more child participants (Table 2). The most commonly reported problems were similar to those reported by adults, and included lack of basic needs, alcohol and drug use, and bad sanitation (e.g., dirty drains, garbage in common spaces). of these 25 problems, 15 were identified by study staff as potentially related to mental health. Similar to the list of adult-reported problems, alcohol and drug use, fighting, and harassment were the second, fifth, and sixth most commonly reported problems, respectively. Others included children being verbally aggressive, disrespecting elders, stealing, engaging in “bad behaviors” (e.g., spending money for school fees on other things), gambling, showing anger (e.g., damaging property, hitting parents), depression, suicide, and sexual abuse.

**Table 2.** Problems of children identified from free-list interviews with children aged 10–17 (n = 40)^a^.

Problem	Frequency (%)
Lack of basic needs (e.g., clean water, toilet facilities, electricity, clothing, space)	39 (98)
*Alcohol & drug use	35 (88)
Squalor/bad sanitation	35 (88)
School-related problems (e.g., children missing school, can’t study, being expelled/failing)	30 (75)
*^+^Fighting	29 (73)
*^+^Harassment (verbal, physical, and sexual harassment)	24 (60)
Bad environment (e.g., excessive noise, parents fighting)	24 (60)
Physical health problems/illness	21 (53)
*Abusing/swearing (verbal aggression)	20 (50)
Child labor	16 (40)
*Disrespecting elders	14 (35)
Lack of child rights	11 (28)
*Stealing	11 (28)
*Bad behaviors (e.g., bad company, troubling others, missing school)	10 (25)
*Discrimination (e.g., gender-based; girls not allowed to study)	8 (20)
*Gambling	7 (18)
*Beating children	7 (18)
*Anger in children (e.g., damaging property, hitting parents)	6 (15)
*^+^Depression	5 (13)
Child marriage	5 (13)
*Suicide	3 (8)
*Sexual abuse	3 (8)
*Inappropriate sexual relationships	3 (8)
Lack of compassion	3 (8)
Unemployment (adults)	3 (8)

*Notes*. ^a^Problems mentioned by three or more respondents. *Potential mental health problems or problems that may have a mental health impact. ^+^Problems that were selected for in-depth key informant interviews.

Overall, there was strong agreement between adult and child reporters on the problems of children living in slums, their prioritization, and those that may have mental health impacts on children in these settings. Ninety-one percent of child-identified problems appeared on the adult list; problems such as child anger, suicide, and sexual abuse were only identified by child participants. Seventy-eight percent of adult-identified problems appeared on the child list, where problems such as poverty, child neglect, and child prostitution were only mentioned by adult participants. The top seven problems were the same on both lists, indicating similar levels of prioritization from child and adult perspectives. Among problems that appeared on both lists, there was 100% agreement on identification of problems that may be related to mental health.

### Key informant results

Three FL problems were selected for in-depth exploration via KI interviews: (1) harassment, (2) fighting, and (3) depression. While “depression” was the term generated in the FL interviews, we use the term “depression and anxiety symptoms” to describe the KI interview results, as this elaborated term more accurately captures the range of internalizing symptoms that were reported in the KI interviews. Tables 3–5 summarize the KI results for each investigated problem, and full data are included in supplemental files.

**Table 3.** Key informant descriptions of “harassment” (n = 33)^a^.

Response	Frequency (%)
***Nature of the problem***	
1. Where harassment takes place (e.g., bathrooms, while filling water, slum lanes, secluded/dark places, social media)	32 (97)
2. Who harasses children (e.g., vagabond boys, drunkards, older girls, maternal & paternal uncles)	30 (91)
3. Abusing/swearing (e.g., crude language, lewd comments)	26 (79)
4. Bothering/troubling (e.g., whistling, lewd gestures, pushing)	24 (73)
5. Touching any part of the child's body (e.g., hands, private parts)	16 (49)
6. Rape	6 (18)
7. Girls tease/harass other girls	4 (12)
8. Forming a group and harassing child / staring in lewd manner	3 (9)
***Causes***	
1. Due to girl being fashionable (e.g., getting dressed up, wearing make-up)	16 (49)
2. When children are alone/remain quiet	12 (36)
3. The girl's face (e.g., beautiful, lively, ugly)	12 (36)
4. Fear (e.g., child harassed more due to not telling anyone about harassment)	9 (27)
5. Due to using substances	9 (27)
6. Learning to harass by watching other people	7 (21)
7. Child with a physical/mental weakness is harassed (e.g., too thin/fat, dark skin, dull mind)	6 (18)
8. Watching adult/pornographic films	5 (15)
9. Boys’ simplicity/innocence	4 (12)
10. Being jealous of each other	4 (12)
***Impact***	
1. Isolation & loneliness	24 (73)
2. Fear	23 (70)
3. Child has negative thoughts (e.g., blaming oneself, thinking no one will help them)	19 (58)
4. Society/community's negative thinking (e.g., thinking harassed child will be a negative influence, blaming harassed child)	18 (55)
5. Family's negative thinking (e.g., feeling dishonored, shame)	17 (52)
6. Irritated behavior	15 (45)
7. Depression	15 (45)
8. Committing/attempting suicide	14 (42)
9. Stop studying	14 (42)
10. Stop eating or going outside	14 (42)
11. Fighting	14 (42)
12. Feeling dishonored/ashamed/bad reputation	12 (36)
13. Problems in the family (e.g., family losing respect, experiencing tension)	12 (36)
14. End of freedom for girls	11 (33)
15. Disturbing thoughts (e.g., about rape, being dishonored)	10 (30)
16. Weak physical condition/bad health	9 (27)
17. Feeling unsafe	7 (21)
18. Crying	7 (21)
19. Other feelings (e.g., sexual arousal, revenge)	7 (21)
20. Feeling angry	7 (21)
21. Early marriage	6 (18)
22. Leaving the house/running away	5 (15)
23. Misunderstanding the community as the enemy	5 (15)
24. Losing interest in doing things	5 (15)
25. Not getting support from the community	4 (12)
26. Not getting help from the police	3 (9)
27. Parents not listening to children	3 (9)
***What people currently do***	
1. Explain/counsel	17 (52)
2. Complain to the police	13 (39)
3. Offer sympathy/console	13 (39)
4. Not do anything/wrongly accuse girl	9 (27)
5. Take the child for an outing	8 (24)
6. Take care of the child (e.g., not leaving child alone)	7 (21)
7. Sending the child away to live with relatives	6 (18)
8. Keeping the child busy	3 (9)
9. Child not being allowed to leave the house	3 (9)
***What should people do***	
1. Explain/counsel	12 (36)
2. Make complaints (e.g., to police, to boy's family)	12 (36)
3. Not leave the child alone	10 (30)
4. Create a good environment for the child	8 (24)
5. Self-defense training	8 (24)
6. Take child for an outing	7 (21)
7. Express love	7 (21)
8. Let the child do the things he/she loves	7 (21)
9. Send child to school	6 (18)
10. Sensitive and good behavior with child	5 (15)
11. Help the child (e.g., support child and keep the friendship)	4 (12)
12. Give consolation/encouragement to the child	4 (12)
13. Threaten the perpetrator	3 (9)
14. Live in harmony with children	3 (9)
15. Keep children away from strangers	3 (9)
16. Find a solution to this problem	3 (9)

*Notes*. ^a^Reported by three or more respondents.

**Table 4.** Key informant descriptions of “fighting” (n = 33)^a^.

Responses	Frequency (%)
**I. Fighting among children**	
***Nature of the problem***	
1. Hitting/beating (e.g., with sticks, stones, knives)	33 (100)
2. Abusing/swearing	31 (93)
3. Breaking/destroying things	21 (63)
4. Bloodshed (e.g., using guns, rods)	20 (60)
5. Teasing/bothering children	16 (48)
6. Falling into bad company (e.g., forming rape gangs)	15 (45)
7. Losing patience/getting angry	13 (39)
8. Fighting in groups	11 (33)
9. Fighting at home and outside	9 (27)
10. Screaming & threatening	7 (21)
***Causes***	
1. Fighting while playing (e.g., pushing/shoving, fighting over play object)	28 (85)
2. Fighting about material things and over small matters	24 (72)
3. Due to seeing older people fight (e.g., parents)	20 (60)
4. Fighting over access to water/toilets	17 (51)
5. Harassing/teasing children	16 (48)
6. When children abuse/speak badly of family members	12 (36)
*7.* Getting encouragement from others to fight (e.g., from parents)	10 (30)
8. Not having fear	7 (21)
9. Due to studies (e.g., fighting with child who is good at their studies)	6 (18)
10. Children not obeying	6 (18)
11. Due to absence of education	4 (12)
12. Due to child not getting their favorite thing	4 (12)
13. Due to using substances	3 (9)
14. Enmity among children	3 (9)
15. Due to influence of films/TV	3 (9)
***Impact***	
1. Negative effect on the child's mind (e.g., feeling despondent/fearful)	28 (85)
2. Irritable behavior	25 (76)
3. Using substances	24 (72)
4. Negative impact on the family (e.g., feeling embarrassment/shame)	23 (70)
5. Not able to study/stops going to school	21 (63)
6. Change in child's disposition (e.g., staying angry, sad, or irritable)	17 (52)
7. Negative impact on the community (e.g., neighbors keep their children away)	17 (52)
8. Negative thoughts/feelings (e.g., thinking one is weak, regret)	16 (48)
9. Loneliness	15 (45)
10. Feelings of revenge/hatred/jealousy	13 (39)
11. Parents stop children from doing things (e.g., going out to play)	13 (39)
12. Enmity between children	13 (39)
13. Bodily injuries	13 (39)
14. Being worried/anxious	12 (36)
15. Life is ruined (e.g., getting arrested)	12 (36)
16. Negative influence on other children	11 (33)
17. Not listening/obeying	11 (33)
18. Feeling shame/dishonor	10 (30)
19. Tension among children & parents	10 (30)
20. Fear is gone (e.g., child not fearing consequences of fighting)	9 (27)
21. Runs away/thrown out of home	9 (27)
22. Stops eating/drinking	8 (24)
23. Fighting becomes a habit	8 (24)
24. Stealing	7 (21)
25. Bad environment in the slum	7 (21)
26. Fear (e.g., children scared of being beaten up again)	6 (18)
27. Depression (e.g., thinking about dying)	6 (18)
28. Not being able to get married	6 (18)
29. Friendships breaking up	5 (15)
30. Losing control of oneself	4 (12)
31. Fighting among elders due to fighting among children	4 (12)
***What people currently do***	
1. Give moral education/explain to children & parents	30 (90)
2. Rescue/intervene in fights	19 (57)
3. Complain to the police	18 (54)
4. Make a good relationship with the child (e.g., parents spend time with child)	12 (36)
5. Keep child away (e.g., from other children who fight)	8 (24)
6. Discipline children (e.g., scolding, hitting)	7 (21)
7. Settlement/compromise between children	5 (15)
8. Make the child focus on their studies	3 (9)
9. Support the child/take their side	3 (9)
10. Not fight at home	3 (9)
***What should people do***	
1. Explain to children/teach them good things	26 (79)
2. Give children a good upbringing/moral education	25 (76)
3. Keep the child busy	19 (57)
4. Keep a good environment in the slum	16 (48)
5. Send child to school	14 (42)
6. Take help from the police	13 (39)
7. Meet the child's basic needs & be loving with the child	11 (33)
8. Keep children away from fighting	7 (21)
9. Take children out for pleasure	6 (18)
10. Give counseling to children	6 (18)
11. Parents should not use abusive language	3 (9)
12. Separate children who are fighting	3 (9)
13. Spend time in NGO	3 (9)
14. Give child feeling of companionship	3 (9)
15. Scold/punish child	3 (9)
16. Involve the child in religious activities	3 (9)
**II. Fighting at home**	
***Nature of the problem***	
1. Hitting (e.g., using hands, sticks, breaking things)	24 (73)
2. Being afraid/sad/crying	16 (48)
3. Not listening, not talking	16 (48)
4. Not studying	14 (42)
5. Not doing daily activities (e.g., eating, sleeping)	10 (30)
6. Bloodshed, injuries	4 (12)
***Causes***	
1. Fighting among people (e.g., relatives)/over things (e.g., property)	33 (100)
2. Lack of money	25 (76)
3. Suspicion/doubt (e.g., between husband & wife)	22 (67)
4. Substance use	21 (64)
5. Abusing (verbal)	20 (61)
6. Fighting over household work	14 (42)
7. Making fun (e.g., backbiting, taunting)	12 (36)
8. Being irritated	8 (24)
9. When marriage arrangement for child doesn’t work out	7 (21)
10. Gambling	7 (21)
11. Bad environment (e.g., no unity among people)	6 (18)
12. Jealousy/enmity	5 (15)
13. Fighting over small things (e.g., toys, TV)	5 (15)
14. Not listening/arguing	4 (12)
15. Not giving time (e.g., to child or to spouse)	3 (9)
16. Making a lot of noise	3 (9)
17. Discrimination	3 (9)
18. Shortage of food/drink	3 (9)
***Impact***	
1. Bad/wrong behavior (e.g., being irritable/angry, not eating, staying quiet, learning swear words)	29 (88)
2. Not feeling interested in studies/not going to school	17 (52)
3. Bad effect on the mind (e.g., running away, suicide)	16 (48)
4. Feeling unsafe/unprotected	14 (42)
5. Feeling embarrassed/shame	11 (33)
6. Thinking a lot about fights at home/feeling troubled	10 (30)
7. Not feeling interested in playing/doing work	10 (30)
8. Learning to fight from elders	8 (24)
9. Not listening to/obeying elders	7 (21)
10. Using substances	7 (21)
11. Feeling sad	7 (21)
12. Abusing (verbal)	5 (15)
13. Hitting (e.g., hitting other children)	5 (15)
14. Having a bad image of one's parents	4 (12)
15. Relationships going bad (e.g., not hosting relatives, spoiled marriage prospects)	4 (12)
***What people currently do***	
1. Explain/give advice	29 (88)
2. Take help (e.g., from police, women's groups)	25 (76)
3. Mediation	11 (33)
4. Education about morals/ethics	4 (12)
5. Make them admit their mistakes, resolve	4 (12)
6. Leave the house and live somewhere else	3 (9)
***What people should do***	
1. Explain/remove suspicion	21 (64)
2. Take help (e.g., from police, village assembly)	17 (52)
3. Give good education about unity	8 (24)
4. Maintain peace in the slum (e.g., explaining importance of love, making laws)	6 (18)
5. Keep people who are fighting apart	5 (15)
6. Increase awareness/counseling (e.g., home visits, plays)	4 (12)
7. Self-defense training	3 (9)
8. Take child out for recreation/send children far away	3 (9)

*Notes*. ^a^Reported by three or more respondents.

**Table 5.** Key informant descriptions of “depression and anxiety symptoms”^+^ (n = 33)^a^.

Response	Frequency (%)
***Signs & symptoms***	
1. *Isolating/feeling lonely/withdrawal	29 (88)
2. ∼Madness/anger	29 (88)
3. *Negative thinking/bad thoughts	27 (82)
4. *Not eating food	22 (67)
5. ^Irritability (e.g., disrespectful of elders)	22 (67)
6. ∼Physical weakness/pain in the mind	15 (45)
7. ^Feeling sad (e.g., “face fallen,” “forgetting the language of love”)	13 (39)
8. ∼Taking things to heart	12 (36)
9. *Not engaging in any entertainment/recreation	11 (33)
10. ∼Thinking too much	9 (27)
11. *Being sluggish/lazy	9 (27)
12. *Not feeling interested/not finding pleasure in doing things	7 (21)
13. Feeling confused	7 (21)
14. Being afraid	6 (18)
15. Throwing tantrums	6 (18)
16. *Not taking care of oneself	6 (18)
17. ^Keeping things in one's heart/hiding things	5 (15)
18. *Not able to sleep	5 (15)
19. *Reduced ability to think and understand	5 (15)
20. *Crying	4 (12)
21. Feeling nervous/anxious	4 (12)
***Causes***	
1. Tension about studies	25 (76)
2. Family tension (e.g., about parents fighting)	24 (73)
3. Basic needs not being met	21 (64)
4. Fighting	16 (48)
5. Not speaking properly/being irritable	14 (42)
6. Taunting child/passing sarcastic remarks	12 (36)
7. Making comparisons to other children	11 (33)
8. Alcohol & drug use (by parents and children)	11 (33)
9. Harassment	11 (33)
10. Tension about the environment	10 (30)
11. Not getting love	9 (27)
12. Tension about goals/career	9 (27)
13. Being married at a young age	8 (24)
14. Sexual harassment (rape)	6 (18)
15. Falling into bad company	5 (15)
16. Another person's death	5 (15)
17. Not telling anyone what happened (e.g., about rape)	4 (12)
18. Pressure at home/oppression by older children	4 (12)
19. Not engaging in entertaining activities	4 (12)
20. Discrimination between boys & girls	3 (9)
21. Mistrust/lack of understanding from family	3 (9)
***Impact***	
1. Negative impact on child's studies (e.g., not studying)	26 (79)
2. Mental tension/stress	24 (73)
3. Negative impact on the child's family (e.g., family is worried, feeling pressured to send child away)	21 (64)
4. Negative impact on the community (e.g., feeling scared child will cause harm, worried child will have a bad influence on others)	19 (58)
5. Bad behaviors (e.g., running away from home, stealing)	17 (52)
6. Using substances	14 (42)
7. Committing suicide	11 (33)
8. Not going to school/college	8 (24)
9. Hurting oneself (e.g., cutting)	7 (21)
10. Career being ruined	7 (21)
11. Threatening to commit suicide	3 (9)
12. Ruining one's life	3 (9)
***What people currently do***	
1. Parents explain child's situation to school teacher	16 (48)
2. Do fun/pleasurable activities with the child	16 (48)
3. Give the child more love and time	14 (42)
4. Take care of the child's needs	13 (39)
5. Create a nice environment/live in harmony	13 (39)
6. Keep the child away from stress	12 (36)
7. Give advice & get treatment	10 (30)
8. Send child far away	8 (24)
9. Share thoughts openly with the child/be the child's friend	5 (15)
10. Counseling at the NGO	4 (12)
11. Providing school/career support	4 (12)
***What people should do***	
1. Provide entertainment/not let the child be alone	25 (76)
2. Parents doing good behaviors with children (e.g., speaking nicely, spending time)	24 (73)
3. Parents should fulfill child's needs	14 (42)
4. Create a good environment/keep good company	10 (30)
5. Parents doing good behaviors in the slum (e.g., maintaining good relationships)	8 (24)
6. Get treatment from a doctor	7 (21)
7. Child should share their thoughts/problems openly	6 (18)
8. Stay away from things that give stress (e.g., fighting, substances)	6 (18)
9. Get love and support from friends	5 (15)
10. Not put pressure on child about studies and their goals	4 (12)
11. Counseling/get help from the NGO	4 (12)
12. Have meetings to talk about children's problems	3 (9)
13. Motivate the child/give skills to face pressure	3 (9)

*Notes*. ^a^Reported by three or more respondents. ^+^Free list term for this problem was “depression.” Study team renamed this “depression and anxiety symptoms” to capture the range of internalizing symptoms described by KI participants. *Depression symptoms that closely overlap with Western conceptualizations and diagnostic criteria for depression. ^Depression symptoms that overlap with Western conceptualizations but use culturally specific descriptors found in the local language Hindi. ∼ Depression symptoms unique to this cultural context.

#### Harassment (“Chedkani”)

*Nature of the problem*. KIs described harassment as a pervasive problem experienced by children living in slums, and noted three common types of harassment: verbal (e.g., swearing at the child, passing lewd comments), physical (e.g., pushing, shoving, pulling the child's clothes), and sexual (e.g., rape, incest) (Table 3). Harassers typically tended to be older boys in the slum, often described as “vagabond, rowdy, or drunkard.” Girls were reported to be harassed more than boys, and harassment took place almost everywhere (e.g., bathrooms, standing in line for water, secluded places in the slum). KIs noted that the high density of the slum and lack of space made it easier for children to be harassed multiple times a day.

*Causes*. Responses from KIs on the causes of harassment among children highlighted underlying gender biases against female victims that were prevalent in these communities. For example, KIs most frequently noted “being fashionable” (e.g., getting dressed up, wearing make-up) and “the girl's face” (e.g., being beautiful, spirited, or lively) as the top two reasons girls were harassed, whereas “boys’ simplicity/innocence” was one of the less commonly reported reasons for boys being harassed. Female children were more frequently blamed for being the victim of harassment than male children. Other frequently reported factors that further maintained or increased harassment were children being left unattended, children not reporting the harassment due to feeling afraid or being threatened by the harassers, substance use by the harasser, and learning to harass by watching other people in the slum do it.

*Impact*. The problem of harassment appeared to have a significant mental health impact on children. The top two most important symptoms observed in children were isolation/loneliness (e.g., staying away, being quiet, feeling sad), and fear (e.g., feeling afraid/anxious, not being able to tell anyone, being hesitant to leave the house). Over half of the respondents noted that children who were harassed often had negative thoughts of self-blame, shame, helplessness, fears of falling sick, and feeling pressure to get married. Feeling depressed and unsafe were also frequently reported as impacts of this problem. Behavioral problems among children who had been harassed included being irritated (e.g., yelling at family members), attempting suicide, not being able to study, not eating/drinking, not going outside, fighting, and crying. Some unique impacts specific to this cultural context that were less frequently reported included “feeling dishonored,” which was described as a deep sense of having brought shame to the family, “end of freedom for girls,” where female children who had been harassed were no longer allowed to leave the house, and early marriage, due to the family's fear that no one would be willing to marry a harassed child as they had been “violated.” The impact of harassment extended beyond the child and led to the child's family having negative thoughts about the family feeling dishonored and ashamed, and the community withdrawing from the child due to concerns about the harassed child having a negative influence on other children in the slum. This individual and collective sense of having brought shame and dishonor to the family appeared to further prevent children from speaking up about harassment, which in turn increased their likelihood of continuing to be harassed.

*Coping strategies.* When asked about what people *currently do* to help children who are being harassed, KIs noted counseling (e.g., giving advice), complaining to the police, and offering sympathy (e.g., comforting, consoling) as the most frequent ways in which people addressed this problem. Almost a third of KIs also reported people often not doing anything about harassment, but instead wrongfully blaming a female child or taunting the child for being harassed. When asked about what people *should do* to help harassed children, in addition to most frequent mentions of counseling and seeking help from the police, KIs most often noted strategies such as not leaving children alone, creating a “good environment” for children (e.g., with less conflict), training children in self-defense, taking children for outings, and parents expressing love towards children.

#### Fighting (“*Ladaee*”)

KIs described witnessing and/or engaging in frequent and violent physical fights as a major problem impacting children living in slums. When describing this in more detail, KIs mentioned two main types of fighting that impacted children living in slums—*fighting among children* (fighting that primarily took place between peers) and *fighting in the home* (fighting between family members, which may or may not involve the child). Study staff asked KIs to report on each type of fighting separately.

### Fighting among children

*Nature of the problem*. KIs most frequently described fighting among children as physically violent acts that involved beating/hitting each other with stones and sticks, tearing each other's clothes, choking each other, and punching, slapping, or biting (Table 4). Of equal importance, while fighting, children also engaged in verbally aggressive behaviors such as arguing, abusing other children and their parents, and screaming/threatening to hurt each other. Children often fought in large groups, which could lead to destruction of property and/or children getting hurt or killed due to the use of guns and knives.

*Causes.* The most commonly reported causes of fights among children were fighting while playing (e.g., disagreements during play often turned into violent fights), fighting over material things and girls, and fighting after watching other adults fight. Less frequently reported causes included fighting over water or toilets (due to shortage of amenities), fighting back when one was being harassed, fighting due to parents or neighbors encouraging children to fight, and fighting when a child spoke badly about another child's family members.

*Impact*. KIs reported significant mental health impacts of fighting among children, which were noted to be even more severe than the physical impacts. These most frequently reported mental health impacts included “effect on the [child's] mind,” which reflected persistent feelings of despondency, irritable behavior, a change in the child's disposition (e.g., staying angry or staying sad for a long period after the fight has ended), negative thoughts (e.g., thinking of oneself as weak), loneliness, feelings of anxiety, tension, shame or dishonor, fear, and depression. Fighting often led to significant physical injuries such as head injuries, dizziness, and broken bones. Other behavioral impacts of fighting among children included children using substances, not going to school, losing interest in studies, getting expelled, disrespecting elders, not eating/drinking, and running away from home. Similar to previous problems, KIs frequently reported a significant impact of fighting on the child's family (e.g., family experiencing “tension,” feeling shame and embarrassment, worrying about the neighbors hating them) and the community (e.g., neighbors keeping their children away).

*Coping strategies*. To stop fights among children, people most frequently provided “moral education” on good behavior, counseled children and their parents, intervened during a fight and separated children who were fighting, and complained to the police. Less frequently reported strategies included keeping the child away (e.g., by sending them to live with relatives), and disciplining children by scolding, hitting, or threatening them. KIs acknowledged that these approaches were often ineffective, and other things that people *should* do to deal with this problem included more counseling for children, especially teaching them “good things and respect,” giving children a “good upbringing” in the form of more moral education, keeping children busy with work, studies, and play, sending children to school, and maintaining a better environment in the slum. Many of these suggestions involved parents and other adult figures in the slum giving children more time and attention.

### Fighting at home

*Nature of the problem*. KI data on *fighting at home* revealed a related set of problems that also had a significant mental health impact on children living in slums (Table 4). Fights at home typically took place between parents, siblings, and relatives. They most frequently involved family members breaking things and hitting each other with objects or their hands and feet, often causing significant physical injuries. During such fights, KIs noted that children would often become very quiet and timid due to feeling afraid, resist talking to anyone, stop studying, and stop participating in their daily activities (e.g., eating, sleeping) due to feeling weak.

*Causes*. Family members most commonly tended to fight about other people (e.g., relatives or children) and property. Other commonly reported causes of *fighting at home* included lack of money (e.g., basic needs not being met), suspicion between a husband and wife (e.g., about a husband's activities if he comes home late after drinking), use of substances, use of abusive language between family members, household work, being taunted/made fun of, or when a marriage proposal for a child was withdrawn.

*Impact.* As a result of *fighting at home*, KIs most frequently reported that children engaged in “bad/wrong behaviors” (e.g., being irritable, staying quiet, crying, not obeying, not eating, and hitting), were not interested in their studies and often didn’t go to school, lost interest in playing, and started using substances themselves. KIs also conceptualized behaviors like running away from home, attempting suicide, and being physically sick as a “bad effect on the child's mind,” as a result of the nature and frequency of fighting at home. Emotional impacts on the child included feeling unsafe, feeling embarrassed, ruminating about the fights at home, and feeling sad. Even if children weren’t directly involved in the conflict at home, one of its important impacts was that children learned to fight by watching their parents fight.

*Coping strategies.* Similar to other problems, people *currently* cope with the problem of *fighting at home* by explaining and giving advice to family members, taking help from the police and slum leaders, and engaging in other forms of mediation. In addition to continuing these activities, KIs frequently noted working to remove suspicion between spouses, providing education about the importance of unity, maintaining peace in the slum, and increasing awareness via counseling, home visits, and street plays as things people *should* do to address the problem of *fighting at home*.

### Depression and anxiety symptoms

*Symptoms.* During KI interviews on the term “depression” from the FLs, KIs described a mix of depression and anxiety symptoms in three categories: 1) symptoms that closely overlapped with Western diagnostic criteria, 2) symptoms that overlapped with Western diagnostic criteria but used culturally specific descriptors found only in the local language, and 3) symptoms that were unique to this context. First, KIs most frequently described a mix of depression and anxiety symptoms that are included in the Diagnostic and Statistical Manual of Mental Disorders (DSM-5) diagnostic criteria for major depressive disorder (MDD) and anxiety disorders (American Psychiatric Association, 2013). The overlap with MDD included symptoms such as *feeling sad*, *loss of interest/pleasure in doing things*, and *poor appetite* (Table 5). The reported symptom of *negative thinking or having bad thoughts* captured other MDD symptoms such as feelings of hopelessness (e.g., “I will always have to tolerate wrong things”), feelings of worthlessness (e.g., “I can’t do anything”), or recurrent thoughts of suicide (e.g., “I wish I were dead”). KIs also reported anxiety-related symptoms such as *feeling anxious/restless*, *aches in the body*, and *feeling afraid*. Some reported symptoms that could be related to depression and/or anxiety included *irritability*, *feeling lethargic*, *reduced ability to concentrate* and *problems with sleep*. The top symptom reported by KIs of *isolating/feeling lonely* may indicate social withdrawal or social impairment due to experiencing depression or anxiety symptoms. Second, for some DSM-5 symptoms, descriptions were uniquely informed by the local culture. For example, *irritability* in a child was primarily associated with behaviors that signaled disrespect, such as speaking rudely to elders, indicating a deviation from the core cultural value of respect for the elderly. “Face remains sad or fallen” or “forgetting the language of love” were unique ways of describing a child who is *feeling sad* in Hindi. Finally, in addition to symptoms that are included in DSM-5 diagnostic criteria for depression and anxiety, unique symptoms that have been found in similar LMIC contexts were also reported frequently (Haroz et al., 2017). The most common was *madness/anger*, described as a child engaging in aggressive behaviors such as fighting, hitting others, breaking things, swearing, and passing sarcastic/critical remarks. Other unique symptoms in this context included *pain in the mind*/*physical weakness* (falling sick, pain in different parts of the body, feeling dizzy), *taking things to heart* (being heavily impacted by what people say), and *thinking too much* (thinking about the same thing over and over again, indicating rumination or uncontrollable worry).

*Causes.* The top two most frequently reported causes of depression and anxiety symptoms in children were tension about education (e.g., tension about failing exams or not getting a good education) and family (e.g., parents fighting and hitting each other). The term “tension” was frequently used by KIs to describe a mental state of being stressed, or to describe a specific type of stressor (e.g., “family tension”). Interestingly, tension about one's education and family were both more frequently rated as causes of depression and anxiety symptoms in children than lack of basic needs such as food or clothing. Other less frequently reported causes included fighting in the slum (e.g., among children), child being taunted (e.g., child mocked for living in a slum), alcohol and drug use by parents and children, and children being harassed.

*Impact.* KIs reported that depression and anxiety symptoms had a severe impact on the child, their family, and the community. The most frequently reported impact on *children* was academic, where a child with these symptoms was not able to concentrate and was not interested in studying. These children also experienced “mental tension” due to stressors in the environment, such as conflict between siblings, domestic violence, or being harassed. Other less frequently reported impacts on children included children engaging in bad behaviors (e.g., running away from home, stealing), using substances, committing suicide, not going to school, engaging in self-harm (e.g., cutting), and not being able to fulfill their career aspirations. KIs also frequently described impacts on the *family* of a depressed-anxious child as the family feeling sad, worrying about getting treatment for the child, worrying about the child running away from home, “thinking badly” about the child (e.g., blaming the child for their symptoms), and feeling pressure from relatives and neighbors to send the child far away. The equally significant impact on *community members* included the community feeling scared that the child will harm others, ignoring and staying away from the child, thinking the child is “bad,” and worrying that the child will have a bad influence on other kids. These results indicated that isolation, which was one of the top reported symptoms, was further maintained by the family's and community's response to a child experiencing depression and anxiety symptoms in the slum.

*Coping strategies*. Among the things that people *currently do* to help children who have depression and anxiety symptoms, KIs most frequently reported strategies such as parents explaining the child's problems to their school teachers, engaging in fun and pleasurable activities with the child (e.g., taking the child for an outing, playing, telling stories), giving the child more love and time, taking care of the child's needs (e.g., providing clothes, books), creating a harmonious environment, and reducing the child's stress. Less frequently reported current strategies included giving advice, seeking treatment, and sending the child far away from the slum (e.g., to the child's village). When asked about what else people *should do* to help children who are experiencing depression and anxiety symptoms, KIs most frequently noted recommendations such as people doing fun activities with the child and not letting the child be alone, and parents doing “good behaviors” with children (e.g., speaking nicely and with love, giving them time and attention). Less commonly reported suggestions included fulfilling the child's basic needs, such as sending them to school, parents doing “good behaviors” in the slum (e.g., maintaining good relationships with people, not fighting), getting treatment from a doctor, having the child openly share their thoughts, staying away from stressors like fighting and using substances, and getting counseling from Asha. These responses suggested that parents modifying their behavior at home and in the slum were potentially important for helping children cope with depression and anxiety symptoms, and that community members were open to considering counseling interventions in these areas to help children manage these symptoms.

## Discussion

The purpose of this study was to understand local conceptualizations of mental health problems among children living in New Delhi slums, with the ultimate goal of identifying and adapting culturally appropriate evidence-based interventions to address these problems. During the FL interviews, over 50% of problems reported by children and adults were potentially related to mental health, indicating that mental health issues were likely to be common in this population. These included mental illnesses such as depression and substance use. They also included common experiences of children such as fighting and harassment, which frequently *led* to other mental health problems. The relative prioritization of these problems in the FL data suggested that even in the face of substantial unmet basic needs, problems related to child mental health are a priority in these communities.

Our KI data indicated that children living in New Delhi slums experience a range of internalizing and externalizing symptoms. The identified mental health problems appear to be highly interconnected, with certain behaviors (e.g., harassment, fighting) often described as both causes *and* impacts of mental health symptoms. This interconnectedness of problems is expected in a population experiencing multiple, chronic stressors and high levels of deprivation and threat (McLaughlin et al., 2014). Our finding that externalizing symptoms (e.g., irritability, explosive anger, physical and verbal aggression) often co-occur with many internalizing symptoms (e.g., sadness, anxiety) has been reported in other diverse populations exposed to similar levels of deprivation and threat (Haroz et al., 2017; Izutsu et al., 2006; Tol et al., 2019). Our results suggest that in New Delhi slums, many of the described proximal causes of mental health problems are chronic stressors (e.g., poverty, sex discrimination), academic pressure, and social pressures (e.g., public shaming by the community).

The presentation of depression and anxiety symptoms among children in this population is consistent with the global mental health literature on depression, which has found DSM-5 diagnostic symptoms as well as other features unique to the local context across depression studies from 77 countries, including India (Aggarwal et al., 2021; Haroz et al., 2017). The features of *social isolation* and *anger* that are not part of DSM-5 diagnostic criteria for MDD were the most frequently reported symptoms in our sample, further supporting the use of measurement tools that include *both* DSM-5 criteria and other features to capture the full manifestation of depression among children living in New Delhi slums. Local descriptions of what “depression” looked like in this context included both depression and anxiety symptoms. This overlap is common, difficult to distinguish, and supported in studies from diverse contexts (Abas & Broadhead, 1997; Bener et al., 2012; Das-Munshi et al., 2008; Haroz et al., 2017; Kessler et al., 2008). Our finding that “tension” was used interchangeably with “depression” and noted as both a cause and impact of depression is similar to findings from other South Asian studies, where “tension” describes a range of emotional and physical symptoms that could be related to depression or anxiety, including sadness, worry, fatigue, weakness, and pain (Karasz et al., 2013; Maitra et al., 2015; Weaver & Karasz, 2022). Another symptom of “thinking too much” reported by our KIs has been linked to ruminative worry found in anxiety and depressive disorders in multiple global studies, including studies conducted in Southeast Asia (Hinton et al., 2012; Kaiser et al., 2015). Taken together, our results indicate that both depression and anxiety symptoms are causes of suffering among children in this population; they both need to be addressed in adapted interventions and included in assessments of intervention effectiveness. The next steps using the DIME methodology will be to: 1) review the literature for existing tools that have been used to measure depression and anxiety symptoms among youth in LMICs including India (e.g., Beck Depression Inventory, Beck Anxiety Inventory, Strengths and Difficulties Questionnaire, Hopkins Symptom Checklist; Yatham et al., 2018); 2) compare available measures with qualitative data from this study to select those that best match how community members describe depression and anxiety symptoms; 3) adapt measures to include culturally specific symptoms or descriptions not typically found in western assessments (e.g., anger, “tension,” “pain in the mind”) to better fit local conceptualizations of symptoms; 4) translate measures to include words and phrases used by community members in the qualitative data to improve acceptability and accuracy of measures; and 5) conduct a validity study with the adapted measures (AMHR, 2011).

In addition to mood problems that emerged as important issues in this study, issues related to conflict and violence were equally salient. The problems of *fighting among children* and *fighting in the home* were complex and frequently noted as both a cause and impact of other mental health-related problems. Notably, the most commonly listed impacts of children exposed to conflict were psychosocial and often severe in nature (e.g., leading to substance abuse). Moreover, KIs frequently reported that children learned physical and verbally aggressive behaviors by witnessing their parents’ conflictual interactions at home. Family conflict has intergenerational effects, with research indicating that children who are exposed to conflict and violence in their family of origin continue to perpetuate this cycle into future generations (Rothenberg et al., 2016, 2017, 2018). This can occur through two pathways. In the first, children exposed to violence frequently become violent themselves and demonstrate multiple *externalizing symptoms* (e.g., defiance, physical aggression). Over time, they learn that acting aggressively is a way to achieve their interpersonal goals, which generalizes to social interactions across the life course and ultimately leads to recurrence of conflict in the next generation of families (Rothenberg et al., 2016). In the second pathway, children exposed to violence become more withdrawn and depressed, demonstrating *internalizing symptoms* (e.g., feeling despondent, having negative ruminative thoughts). They withdraw to avoid adverse consequences of the family conflict, which may generalize as a coping strategy for managing conflict in other social interactions over time. This withdrawal-depressed interaction style often persists into adulthood and can lead to ongoing conflict in their new families (Rothenberg et al., 2018). Rothenberg and colleagues (2018) also found that these processes are developmentally specific, where externalizing symptoms by age 15 and internalizing symptoms during ages 14 to 21 were both strong mediators of the association between conflict across two generations. Given the high exposure to conflict and violence among children in Delhi slums, along with how early these pathways may be crystallized, targeting internalizing and externalizing symptoms in childhood is critical to breaking the possible intergenerational transmission of conflict in these communities.

The bidirectional relationship between academic stress or poor academic performance and mental health problems among children is notable in this study. This relationship has been established in multiple U.S. and LMIC studies, and is particularly salient in Asian and South Asian cultures (Ahn & Baek, 2013; Dunne et al., 2010; Karasz et al., 2019; McLeod et al., 2012; Valdez et al., 2011). In southern India, adolescents experiencing academic stress had a 2.4 times higher risk of developing depression compared to adolescents not experiencing academic stress (Jayanthi et al., 2015). In our study, “tension” or stress about academic performance was reported as the leading cause of depressive and anxiety symptoms among children living in slums. Relatedly, KIs reported that *all* the mental health-related problems explored in this study had negative academic impacts, such as the child not going to school or not being able to concentrate on schoolwork. In a context where academic achievement is a deep-rooted cultural value and viewed as the path out of poverty, this impact is significant and debilitating. Once children start falling behind on their academic milestones, parental pressure and public shaming around their academic failures continue to maintain their depression and anxiety symptoms. Intervening on mental health problems would not only improve psychological well-being among children but also has the potential to break this cycle by improving academic outcomes among youth living in New Delhi slums.

KIs reported that current strategies employed to address these problems had mixed results. Some of the most frequently mentioned strategies (e.g., providing moral education to reduce fighting) were also reported to be ineffective, further warranting the need for additional support to address these problems. Given the presence of chronic stressors in this environment (e.g., poverty, gender discrimination, witnessing conflict and violence, academic pressure), along with deep embeddedness in family and community relationships that is reflective of collectivist cultures, one approach for Asha is to consider preventative interventions that bolster resilience among youth living in slums. In his work, Dr. Michael Ungar (2008) defines resilience as follows:

In the context of exposure to significant adversity, resilience is both the capacity of individuals to navigate their way to the psychological, social, cultural, and physical resources that sustain their well-being, and their capacity individually and collectively to negotiate for these resources to be provided in culturally meaningful ways. (p. 225)

Dr. Ungar and colleagues developed the R2 program, a multisystemic approach to building resilience by strengthening two types of factors: 1) rugged protective factors that are changeable internal qualities (e.g., gratitude, self-esteem, optimism, mindfulness), and 2) resources required to overcome exposure to adverse psychosocial factors (e.g., supportive relationships, powerful identity, experiences of control) (Ungar & Jefferies, 2021). Their research has shown that both aspects of resilience are necessary for positive outcomes in a context of chronic stress and adversity (Ungar & Theron, 2020). This framework can be used to assess and enhance locally based interventions that may address the mental health problems identified in this study from a more systemic, resilience-based perspective. For example, Asha currently has community centers in each slum through which they deploy holistic community-based programs in health care, education, environmental improvement, and financial inclusion. Empowering community members to lead these programs is at the heart of Asha's mission. Asha has a longstanding Youth Empowerment Program, where children meet regularly in *Bal Mandals* or children's groups at their local slum community center. These groups offer activities to foster core Asha values, including gratitude, optimism, affirmation, joy, and non-violence. Bal Mandal youth also lead and participate in multiple health- and education-based interventions that further nurture their self-identity and help build supportive relationships in their environment. There is already significant overlap between R2 and Asha's Youth Empowerment Program. Using the R2 approach to enhance Asha's existing Youth Empowerment Program would be a natural next step to target risk and protective factors of mental health among youth living in slums. Based on this study's data, relevant outcomes of a resilience-building program could include improved self-esteem among children who are harassed, higher rates of school engagement, fewer delinquent behaviors, or fewer rates of violence towards children. In addition, contextually specific protective factors that may be important targets include improved healthy relationships between youth and caregivers, family cohesion, and increased control in the youth's life.

Interventions focused on treating mental health symptoms among youth experiencing clinically significant levels of distress or impairment also warrant consideration. The frequent mention of the word “counseling” in our data suggests that psychotherapy interventions may be acceptable in this community. Multiple randomized controlled trials have established evidence for the feasibility and effectiveness of cognitive behavioral therapy (CBT) in reducing depression and anxiety symptoms in similar LMIC contexts (Betancourt et al., 2014; Bolton et al., 2007; Dorsey et al., 2020; Murray et al., 2015; Patel et al., 2010). In this study, we found that many strategies currently employed by the community to address these mental health-related problems are consistent with existing, evidence-based CBT interventions. For example, KIs frequently indicated helping children to engage in pleasurable activities to target depression symptoms, which is a core element of Behavioral Activation for depression (McCauley et al., 2015). KIs also frequently suggested that parents should change their own behaviors as a strategy to positively impact their child's behaviors. This is a foundational principle of multiple parenting interventions (e.g., Parent–Child Interaction Therapy, Brinkmeyer & Eyberg, 2003; Parent Management Training, Forgatch & Patterson, 2010), which have been culturally adapted in LMICs and shown to be effective in decreasing child externalizing symptoms, reducing family conflict, and improving parent–child relationships (Baumann et al., 2014; Burkey et al., 2018; Knerr, Gardner & Cluver, 2013; Mejia et al., 2012). Given the overlap and co-morbidity between mental health-related problems in these communities, transdiagnostic treatments that are flexible and can address multiple symptoms of anxiety, depression, trauma, and behavioral problems among children are likely to be the most beneficial (Martin et al., 2018). Some examples of transdiagnostic interventions that have been adapted and successfully delivered in LMICs include the Common Elements Treatment Approach (CETA; Bolton et al., 2014; Murray et al., 2014, 2018; Weiss et al., 2015) and Problem Management Plus (PM+; Dawson et al., 2015; Rahman et al., 2016; Sijbrandij et al., 2016). These treatments hold promise for being able to target the range of interconnected internalizing and externalizing symptoms present in this context.

One of the unique strengths of this study is the use of the DIME methodology, which centers community perspectives on the mental health needs of the population and emphasizes building local capacity to conduct and participate in the research. Asha staff members were trained in several core research skills, including designing instruments, recruiting and consenting participants, conducting semi-structured interviews, and analyzing qualitative data. Building mental health research capacity in LMICs by focusing on training in core, transferrable research skills is critical for improving equity in global mental health research (Thornicroft et al., 2012). It reduces reliance on western researchers, creating a more equitable partnership between U.S.-based and LMIC-based organizations. In addition, it ensures that research methods are adapted to be culturally appropriate and therefore more acceptable to the target population. For example, Asha staff were integrally involved in designing the interview guide and phrasing questions in a way that would be easily understood by participants. Using interviewers who spoke Hindi and were highly trusted by the target population helped us meet our recruitment goals and allowed us to collect high-quality interview data. It allowed community members to openly discuss mental health topics that carry high levels of stigma in this population. Having the Asha team lead data analyses was critical in ensuring that the data were being accurately interpreted and in identifying gaps in our understanding. Finally, Asha staff were equipped with qualitative research skills that can be applied to conduct needs assessments and evaluate other programs being implemented by the organization. Taken together, this study highlights the many benefits of upskilling local capacity for the target population and the partner organization, and the validity of research findings. Future studies that use the DIME methodology with this population (e.g., development and validity testing of clinical tools, piloting mental health interventions) will continue to have Asha staff at the frontline of leading and participating in the research, with an ongoing emphasis on building individual- and organizational-level research capacity.

## Limitations

The two slums for this study were chosen by Asha as they would be the first two sites where Asha could most feasibly implement a mental health intervention due to existing resources and longstanding relationships with community members. However, since local culture differs across slum regions in India, the generalizability of these findings remains unknown. Second, while convenience sampling was an appropriate approach for the study aims, the frequency of responses cannot be assumed to represent the prevalence of a given problem, and this method of sampling does not ensure representativeness of the population. Rather than interpreting the numbers as absolute frequencies, this method only indicates the relative importance of problems from the respondents’ perspectives. Another limitation of this study is the use of handwritten notes by the interviewers instead of transcribing recorded interviews. We decided not to record interviews to maintain respondents’ privacy and increase the efficiency of our analyses. While having interviewers work in pairs and combine their notes into one single record was done to increase accuracy, this approach was unlikely to reach the accuracy level of transcribed interviews. Finally, this rapid approach only allowed for follow-up on three potential mental health-related problems. For programmatic reasons given by Asha stakeholders, we did not examine the frequently reported problem of alcohol and drug use in more detail. However, its frequent mention in the FLs and its designation as a cause and impact of other mental health problems suggests that this is a significant problem among children in this community. More detailed information about alcohol and drug use would be critical for developing a holistic mental health care program for children living in New Delhi slums.

## Conclusion

To our knowledge, this is the first qualitative study to assess local perceptions of the mental health needs of children living in New Delhi slums. Our findings suggest that children living in these impoverished neighborhoods have many adverse experiences that lead to internalizing and externalizing symptoms consistent with depression, anxiety, and conduct problems. Community perspectives indicate that even in the face of several unmet basic needs, mental health problems are important to the community. Our data underscored the interconnectedness of these mental health problems, their causes, and their impact, suggesting the need for interventions that can break cycles of violence or harassment that maintain psychosocial distress among children. Our findings also indicate that psychotherapy treatments are likely to be acceptable in this community, and should target changes in child mental health symptoms, parent behaviors, and parent–child interactions. If implemented and effective, these interventions can have a substantial, positive impact on children's mental health, academic outcomes, and family functioning. This study was the first part of a series of activities that will be conducted to identify and adapt specific mental health interventions for children that could be provided by lay workers and would be acceptable to local community members. These findings will inform subsequent assessment and intervention efforts to reduce the burden of mental illness among children living in urban slums in India.

## Supplemental Material

sj-pdf-1-tps-10.1177_13634615231202098 - Supplemental material for A qualitative study of mental health problems among children living in New Delhi slums Supplemental material, sj-pdf-1-tps-10.1177_13634615231202098 for A qualitative study of mental health problems among children living in New Delhi slums by Prerna Martin, Emily E. Haroz, Catherine Lee, Paul Bolton, Kiran Martin, Rosemary Meza, Elizabeth McCarthy and Shannon Dorsey in Transcultural Psychiatry

sj-pdf-2-tps-10.1177_13634615231202098 - Supplemental material for A qualitative study of mental health problems among children living in New Delhi slums Supplemental material, sj-pdf-2-tps-10.1177_13634615231202098 for A qualitative study of mental health problems among children living in New Delhi slums by Prerna Martin, Emily E. Haroz, Catherine Lee, Paul Bolton, Kiran Martin, Rosemary Meza, Elizabeth McCarthy and Shannon Dorsey in Transcultural Psychiatry

sj-pdf-3-tps-10.1177_13634615231202098 - Supplemental material for A qualitative study of mental health problems among children living in New Delhi slums Supplemental material, sj-pdf-3-tps-10.1177_13634615231202098 for A qualitative study of mental health problems among children living in New Delhi slums by Prerna Martin, Emily E. Haroz, Catherine Lee, Paul Bolton, Kiran Martin, Rosemary Meza, Elizabeth McCarthy and Shannon Dorsey in Transcultural Psychiatry

sj-pdf-4-tps-10.1177_13634615231202098 - Supplemental material for A qualitative study of mental health problems among children living in New Delhi slums Supplemental material, sj-pdf-4-tps-10.1177_13634615231202098 for A qualitative study of mental health problems among children living in New Delhi slums by Prerna Martin, Emily E. Haroz, Catherine Lee, Paul Bolton, Kiran Martin, Rosemary Meza, Elizabeth McCarthy and Shannon Dorsey in Transcultural Psychiatry

## Notes

1. The term “slum” was the preferred term used by our local partner organization (Asha) and target population members to describe their communities.
