# Supplementary material for: A qualitative study of mental health problems among children living in New Delhi slums
Source: Transcult Psychiatry. 2024 Feb 23;61(4):533–56. doi: 10.1177/13634615231202098 (PMC11538746; doi:10.1177/13634615231202098)
Supplement: sj-pdf-1-tps-10.1177_13634615231202098 - Supplemental material for A qualitative study of mental health problems among children living in New Delhi slums [file sj-pdf-1-tps-10.1177_13634615231202098.pdf]

Supplemental File: Key Informant Data

Supplemental Table 1. Harassment ($N = 33$)[a]

Cover Term	*Included Terms*	*Frequency (%)*
Nature of the Problem		
1. Where harassment takes place	Bathroom, on the road, while filling water, in the slum lanes, at school, in secluded places, in buses, at bus stops, outside tuition class [i.e., extra after-school class], outside school, in the metro, at weddings, at offices, in parks, in the market, in dark places, social media (Facebook, WhatsApp, google)	32 (97)
2. Who harasses children	Vagabond/rowdy boys, single boys, drunkards, unclaimed/orphaned boys, people who play the big drums, older girls, younger kids, family members (maternal & paternal uncles)	30 (91)
3. Abusing/swearing	Swearing or using crude language, abusing child's parents, saying "I love you", sending "bad" text messages, passing unpleasant/lewd comments	26 (79)
4. Bothering/troubling	Throwing garbage, whistling, making lewd gestures, pushing & shoving, writing love letters, singing songs	24 (73)
5. Touching any part of the child's body	Holding hands, pulling/touching clothes, touching private parts, kissing	16 (49)
6. Rape	Force oneself [sexually, on child]	6 (18)
7. Girls tease/harass other girls	Pulling ponytail, pushing/shoving	4 (12)
8. Forming a group and harassing child	Forming a group and harassing [child], harassing while standing in a group, staring in lewd manner	3 (9)
Causes		
1. Due to being fashionable	Because a girl laughs [implying she is encouraging the boy], the way she dresses, getting dressed up, wearing make-up, on seeing the girl's body	16 (49)
2. When children are alone/remain quiet	When children are alone [unsupervised], because children are quiet	12 (36)
3. The girl's face	Beautiful girls, spirited/lively, stylish, her character, ugliness	12 (36)
4. Fear	Due to being threatened, not telling [anyone] that they are being harassed, not telling family [that they are being harassed]	9 (27)
5. Due to using substances	[Harass children] after drinking alcohol, smoking cigarettes	9 (27)
6. Learning to harass by watching other people	[Learn to harass by] watching each other, older people, films, parents and the environment	7 (21)
7. Physical/mental weakness	On being too thin/fat, teased for being dark in color, dull mind [not intelligent], harass "mad" children	6 (18)

8. Watching TV	Watching adult/pornographic films	5 (15)
9. Boys' simplicity/innocence	Innocent/simple & well behaved [i.e. moral] boys, who are good in their studies, with innocent boys	4 (12)
10. Being jealous of each other	Due to being enemies, being jealous, harassing after threatening [each other]	4 (12)
Impact		
1. Isolation & loneliness	Sad, not being with anyone [not interacting, not staying in people's company], not talking to anyone, being lost in one's thoughts	24 (73)
2. Fear	Feeling anxious, timid/hesitant to leave the house, go to school, not being able to tell anyone	23 (70)
3. Child has negative thoughts	Keep thinking that no one will help them, blaming oneself, feeling ashamed, fear of falling sick, thoughts that they should get married, heart superstition	19 (58)
4. Society/community's negative thinking	Worry that the child who has been harassed might negatively influence their children, child being blamed by relatives [for the harassment], not talking to child who has been harassed	18 (55)
5. Family's negative thinking	Family wondering why they are poor, how the girl child will get married, feeling dishonored, feeling shame	17 (52)
6. Irritated behavior	Yelling at family, feeling restless, not talking to friends properly due to jealousy	15 (45)
7. Depression	Losing control of oneself, feeling tense, feeling strained	15 (45)
8. Suicide	Committing/attempting to commit suicide	14 (42)
9. Stop studying	Not able to finish studying, lost friendships, not being able to study	14 (42)
10. Stop eating or going outside	Not roaming outside [for pleasure], stop talking, not going outside the house	14 (42)
11. Fighting	Fights among each other, children and elders	14 (42)
12. Dishonored/bad reputation	Experiencing a sense of shame, feeling disrespected, feeling bad	12 (36)
13. Problems in the family	Family losing their respect, family coming into tension, family being dishonored	12 (36)
14. End of freedom for girls	Restricted from going in and out, restricted from going to school and tuition [i.e., extra after-school class]	11 (33)
15. Disturbing thoughts	Thoughts about rape, about not talking to anyone, about falling sick, about getting married, about being dishonored	10 (30)

16. Weak physical condition/bad health	Face becomes weak, weakness, not being able to sleep, suffocating on the inside, life being ruined	9 (27)
17. Feeling unsafe	Nobody to help them, nobody to support/be with them, nobody to listen to them	7 (21)
18. Crying	Girls crying due to their troubles	7 (21)
19. Other feelings	Feeling sexually aroused, feeling the desire to make friends with boys (after watching adult films), after watching parents, feelings of revenge	7 (21)
20. Feeling angry	Screaming, tearing one's clothes	7 (21)
21. Early marriage	Being married off early	6 (18)
22. Leaving the house	Leaving the house [running away], or going someplace else, feeling upset/bothered	5 (15)
23. Misunderstanding the community	Thinking that the community/society as a whole is the enemy, misunderstanding/thinking badly of other boys [who do not engage in harassment], not liking sympathy from community	5 (15)
24. Losing interest in doing things	Lose interest in doing work at home, doing work outside, lose interest in doing anything	5 (15)
25. Not getting support from the community	Not getting support from the community/society, people in the community blame the girl [for harassment], not being allowed to play	4 (12)
26. Not getting help from the police	Police takes them away	3 (9)
27. Parents not listening	Parents not listening to children, hiding things	3 (9)
What people currently do		
1. Explain/counsel	Explaining, giving advice, helping, supporting child and parents	17 (52)
2. Complain to the police	Filing a complaint with the police, asking for police protection, there should be a police stand [near the slum]	13 (39)
3. Offer sympathy	To offer sympathy, comfort/console, to support, protect from dishonor	13 (39)
4. Not do anything	Some people don't do anything, wrongly accusing the girl, disrespecting, taunting child/passing sarcastic remarks	9 (27)
5. Take the child for an outing	Take the child for an outing, for pleasure, roaming around	8 (24)
6. Taking care of child	Mom leaving work to take care of girl, not leaving girl alone, speaking [to child] with love	7 (21)

7. Sending the child away	Sending child to the relatives, leaving the home	6 (18)
8. Keeping the child busy	Keep [the child] busy in other things, making them forget [what happened]	3 (9)
9. Child not being allowed to leave the house	Child not being allowed to leave the house	3 (9)
What should people do		
1. Explain/counsel	Should explain/counsel [the child], should awaken [child's] self-confidence, should support the child's parents	12 (36)
2. Make complaints	Getting police protection, filing complaints, complaining to the boy’s family, taking necessary steps to send [harasser] to jail	12 (36)
3. Not leaving the child alone	Not leaving children alone, not letting them go outside, keeping them with you, spending time with children	10 (30)
4. Create a good environment	Should give the child a good environment	8 (24)
5. Self-defense training	Self-defense training, judo, karate, make children self-dependent	8 (24)
6. Take child for an outing	Should take the child for an outing [take outside, for pleasure, roaming around]	7 (21)
7. Expressing love	Should speak with love, should keep the child happy	7 (21)
8. Letting the child do the things he/she loves	Letting the child do things he/she is interested in, giving them clothes	7 (21)
9. Send child to school	Should send [child] to school, make them study, give them education	6 (18)
10. Sensitive and good behavior with child	Sensitive and good behavior with child	5 (15)
11. Help the child	Should help the child who has been harassed, support them, keep the friendship	4 (12)
12. Give consolation to the child	To provide consolation, encouragement, support	4 (12)
13. Threaten the perpetrator	Threaten the perpetrator, beat them up	3 (9)
14. Live in harmony with children	Living in harmony with children, keeping children happy, celebrating birthdays, speaking to them with love	3 (9)
15. Keeping [children] away from strangers	Keeping [children] away from strangers	3 (9)
16. Finding a solution to this problem	Should find a solution, should understand the problem and child's troubles	3 (9)

[a] Reported by three or more respondents.
