# Supplementary material for: A qualitative study of mental health problems among children living in New Delhi slums
Source: Transcult Psychiatry. 2024 Feb 23;61(4):533–56. doi: 10.1177/13634615231202098 (PMC11538746; doi:10.1177/13634615231202098)
Supplement: sj-pdf-2-tps-10.1177_13634615231202098 - Supplemental material for A qualitative study of mental health problems among children living in New Delhi slums [file sj-pdf-2-tps-10.1177_13634615231202098.pdf]

Supplemental Table 2. Fighting among children ($N = 33$)[a]

Cover Term	Included Terms	Frequency (%)
Nature of the problem		
1. Hitting/beating	Hitting with stones, hitting/beating up, picking up things and hitting with them, hitting with sticks, hitting with utensils, tearing clothes, hitting with stones while walking, choking, hitting with a knife, hitting the head, punching, slapping, biting, cutting with a blade	33 (100)
2. Abusing/swearing	Abusing (verbal; swear words), abusing parents, to damn, to argue	31 (93)
3. Breaking/destroying things	Breaking/destroying things, breaking mirrors, breaking things with stones	21 (63)
4. Bloodshed	Using knives, rods, and guns while fighting, bloodshed, killing each other	20 (60)
5. Teasing/bothering children	Teasing/harassing children while they're walking, pulling hair, scratching (with nails), teasing children about studies, calling other children illiterate, saying provocative things, jealousy [among children]	16 (48)
6. Falling into bad company	Falling to bad company, ruining one's life, forming rape gangs, using other people's bodies	15 (45)
7. Losing patience/getting angry	[Children] getting angry, losing patience, losing one's calm, enmity	13 (39)
8. Fighting in groups	Fighting with groups, calling friends and fighting	11 (33)
9. Fighting at home and outside	Fighting at home and outside, fighting among siblings	9 (27)
10. Screaming & threatening	Threatening (e.g. I'll see you outside [to fight])	7 (21)
Causes		
1. Fighting while playing	While playing, joking or riding a bike, when [children are] pushed/shoved, when you don't give them things that they like to play with, when pulling their hair, due to lack of space	28 (85)
2. Fighting about material things and over small matters	Fighting over small matters, fighting about girls, about TV, when [someone] asks [them to] return something they own, pulling hair, hitting with pens, pencils, ink, marbles when fighting over small things, fighting over food	24 (72)
3. Due to seeing older people fight	Watching parents fight and learning from them	20 (60)
4. Fighting over access to water/toilets	While bathing in the bathroom, while filling water, fighting over the toilets, fighting about garbage	17 (51)

5.	Harassing/teasing children	Being harassed while walking, joking/making fun of, speaking rubbish/nonsense, to create trouble, calling the child a black crow [because they have dark skin], harassing girls, touching girls, bumping up against girls, harassing younger children – all these things lead to fights	16 (48)
6.	When children abuse/speak badly of family members	Due to abusing [child's] mother and father, swearing about the sister, using mother and sister swear words to call someone, swearing at the child, saying the child's mother or sister is dancing [local insult implying they are dancing provocatively], swearing/abusing, threatening to kill parents	12 (36)
7.	Getting encouragement from others to fight	Parents encourage children to fight, taking support from parents to fight, neighbors encourage children to fight	10 (30)
8.	Not having fear	Fear goes away, [children] enjoy fighting	7 (21)
9.	Due to studies	Fight with a child who is good in their studies, fighting while going to school, fighting to go to tuition [i.e., extra after-school class], fighting due to not studying	6 (18)
10.	Children not obeying	Not being able to go where they want due to not obeying parents	6 (18)
11.	Due to absence of education	Due to the absence of education	4 (12)
12.	Due to child not getting their favorite thing	Fighting due to child not getting their favorite thing, due to not being taken care of, being obstinate	4 (12)
13.	Due to using substances	Alcohol, cigarettes, marijuana	3 (9)
14.	Enmity among children	Old enemies	3 (9)
15.	Due to influence of films/TV	Fighting after watching films	3 (9)
Impact			
1.	Negative effect on the child's mind	Fighting has a negative effect on the [child's] mind, feeling downcast or despondent, feeling fearful	28 (85)
2.	Irritable behavior	Thinking about hitting, feeling small, speaking in anger, abusing, pride/arrogance	25 (76)
3.	Using substances	Using substances upon losing fight, going on the wrong path, gambling, drinking alcohol, smoking cigarettes, marijuana, stimulants, smelling eraser fluid	24 (72)
4.	Negative impact on the family	Children become ready to leave the house, [family] coming into tension, embarrassment, worrying that neighbors will start hating them, family is troubled and experiences shame	23 (70)
5.	Not able to study	Stop going to school, lose interest in studies, not able to study, leave school, failing in school, being expelled from school	21 (63)

6.	Change in child's disposition	Change in child's mood, staying angry, staying sad, irritability, being obstinate, not eating food	17 (52)
7.	Negative impact on the community	Neighbors keep their children away, coming into tension, making life miserable, ignoring the child	17 (52)
8.	Negative thoughts/feelings	Child's negative thoughts, thinking badly [for e.g., why did I fight], thinking of oneself as strong, thinking one is weak, feeling regret	16 (48)
9.	Loneliness	Not feeling interested in work, stop talking to one another	15 (45)
10.	Feelings of revenge	Child thinks about taking revenge, keeping hatred in the heart, stop talking, feelings of jealousy	13 (39)
11.	Parents stopping children from doing things	[Parents] stop children from doing things, don't let them play, don't leave them alone	13 (39)
12.	Enmity between children	Enmity between children	13 (39)
13.	Bodily injuries	Bleeding, head splitting open [bleeding], feeling dizzy, hands breaking, becoming weak, clothes tearing, becoming disabled after fights	13 (39)
14.	Being worried/anxious	Worrying about family, family's thoughts, older people stop speaking [to the child], feeling upset/disturbed	12 (36)
15.	Life is ruined	Getting arrested due to fighting, going to jail, life is ruined	12 (36)
16.	Negative influence on other children	Children who fight have a negative influence on other children	11 (33)
17.	Not listening/obeying	Not respecting, not listening to anyone	11 (33)
18.	Feeling shame/dishonor	Feeling embarrassed, feeling dishonored/insulted, feeling small	10 (30)
19.	Tension among children & parents	Not being able to sleep (due to tension), children come into tension, parents come into tension	10 (30)
20.	Fear is gone	Children fight more when they are no longer afraid, fighting become part of their behavior, children not fearing consequences/what will happen after fighting	9 (27)
21.	Run away	Children leave their homes, run away, children are thrown out of their homes, children are separated and live away from the family	9 (27)
22.	Stop eating/drinking	Children stop eating/drinking	8 (24)
23.	Fighting becomes a habit	Fighting becomes a habit for children, always thinking about fighting	8 (24)
24.	Stealing	Children snatch and steal	7 (21)

25.	Bad environment in the slum	Fighting spoils the environment in the slums, not wearing proper clothes	7 (21)
26.	Fear	Children are scared that they will be beaten up again, feeling upset/disturbed	6 (18)
27.	Depression	Coming into depression, staying troubled/upset/disturbed, thinking about dying, impact on the mind	6 (18)
28.	Not being able to get married	Not caring about one's future, future gets ruined, child can't get married	6 (18)
29.	Friendships breaking	Friendships break, become enemies	5 (15)
30.	Losing control of oneself	Losing control of oneself, losing one's senses	4 (12)
31.	Fighting among elders due to fighting among children	Fighting among elders due to fighting among children	4 (12)
What people currently do			
1.	Giving moral education/explaining to children & parents	Explaining to children, explaining with love, explaining to parents, explaining through neighbors, giving moral education	30 (90)
2.	Rescuing/intervening	Rescuing children/intervening in the middle, separating the children who are fighting, breaking up the fight	19 (57)
3.	Complaining to the police	Calling the police, getting advice from the slum president, getting help from the police	18 (54)
4.	Making a good relationship with the child	Parents make a good relationship with the child, spending time with the child, taking them to a party, going for entertainment, not leaving the child alone	12 (36)
5.	Keeping child away	Keep child away from other children who fight, not letting child play, take child far away and explain, sending child away to live with relatives, sending child away to a hostel	8 (24)
6.	Disciplining children	Scolding children, hitting children, slapping children, locking children in a room, threatening children	7 (21)
7.	Settlement	Getting children to come to a settlement/compromising, embracing each other [as a sign of resolving conflict]	5 (15)
8.	Making the child focus on studies	Making the child focus on their studies, sending them to school	3 (9)
9.	Supporting the child/taking their side	Supporting the child/taking their side	3 (9)
10.	Not fighting at home	Not fighting at home	3 (9)
What should people do			

1. Should explain to children	Should explain to children, teach them good things, teach them to respect, explain through a counselor, give children sex education in school	26 (79)
2. Giving children a good upbringing	Giving children a good upbringing, educating children, giving them moral education, not restricting them too much	25 (76)
3. Keeping the child busy	Keeping child busy in other work, studies, in playing, explaining to the parents to give time to the child, keeping the child busy in household work	19 (57)
4. Keeping a good environment in the slum	Keep a good environment in the slum, children should make good friends	16 (48)
5. Sending child to school	Should send the child to school, send for tuitions (extra classes), send child to school regularly, pay attention to child's studies	14 (42)
6. Taking help from the police	Complain to the police, take help from police, there should be a police stand in the slum, conduct rallies, take help from president	13 (39)
7. Meeting the child's needs	Meeting the child's basic needs, giving the child their favorite thing, giving child food/drink, giving child things of necessity, being loving with the child	11 (33)
8. Keep children away from fighting	Keep children away from fighting, keep children away from girls	7 (21)
9. Taking children out	Taking children out [for pleasure]	6 (18)
10. Should give counseling to children	Should give counseling to children	6 (18)
11. Parents should not use abusive language	Parents should not use abusive language	3 (9)
12. Separating the children	Should separate both the children [who are fighting]	3 (9)
13. Spending time in NGO	Doing courses	3 (9)
14. Give child feeling of companionship	Giving child feeling of companionship [making them feel like you are friends], living in harmony	3 (9)
15. Scold/punish children	Should scold the child, keep them in fear, should punish the child	3 (9)
16. Involve the child in religious activities	Involve the child in religious activities, going to Satsang [spiritual discourse/sacred gathering], watching Aastha channel on TV, etc.	3 (9)

[a] Reported by three or more respondents.
