# Supplementary material for: A qualitative study of mental health problems among children living in New Delhi slums
Source: Transcult Psychiatry. 2024 Feb 23;61(4):533–56. doi: 10.1177/13634615231202098 (PMC11538746; doi:10.1177/13634615231202098)
Supplement: sj-pdf-3-tps-10.1177_13634615231202098 - Supplemental material for A qualitative study of mental health problems among children living in New Delhi slums [file sj-pdf-3-tps-10.1177_13634615231202098.pdf]

Supplemental Table 3. Fighting at home (N = 33)[a]

Cover Term	*Included Terms*	*Frequency (%)*
Nature of the problem		
1. Hitting	Anger, being a fighter, breaking things, using long sticks/poles (for hitting), using with hands/feet (for hitting), becoming stubborn	24 (73)
2. Being afraid	Becoming timid, staying quiet, moony, crying, feeling stressed/tense, lack of courage, sad	16 (48)
3. Not listening, not talking	Not listening [to what others are saying to do], not talking	16 (48)
4. Not studying	Not feeling interesting in studies, feeling bad, strolling around like a vagabond, failing in school	14 (42)
5. Not doing daily activities	Not eating, not sleeping, feeling weak	10 (30)
6. Bloodshed, injuries	Injuring others, head exploding [bleeding], blood shed	4 (12)
Causes		
1. Fighting among people/over things	Fighting over children, between children, between friends, throwing pens/pencils/books, fighting about relatives, fighting between relatives (e.g., between mother and daughter-in-law, siblings, uncles, grandparents, father-son), fighting about property, fighting while filling water, fighting over garbage/drains, fighting in the home due to fighting outside, others' fights happening in the home	33 (100)
2. Lack of money	Fighting about expenses, basic needs not being met, [fighting over] clothes, food, books, lack of money, blaming, fighting due to not working (and therefore not earning)	25 (76)
3. Suspicion/doubt	Fighting between husband and wife, fighting due to doubting/being suspicious, due to husband coming home after drinking, due to husband coming home late, brothers doubt their sisters	22 (67)
4. Substance use	Due to alcohol, cigarettes, tobacco, tobacco mixed with areca nut, catechu, paraffin wax, slaked lime (chewed)	21 (64)
5. Abusing (verbal)	Abusing, being disrespectful, screaming swear words (about mother and sister)	20 (61)
6. Fighting over household work	Fighting over household work	14 (42)
7. Due to making fun	Backbiting, destroying the peace (of neighbors), jinxed/wretched home, complaining, instigating, taunting, making fun	12 (36)
8. Due to being irritated	Watching the spectacle (during fights), irritated behavior	8 (24)

9. When marriage arrangement for child doesn't work out	Fighting about child's marriage, marriage proposal falling through	7 (21)
10. Gambling	Due to gambling, selling household items	7 (21)
11. Bad environment	No unity, wrong perception/misunderstanding among people	6 (18)
12. Jealousy	Jealous, enmity, to threaten	5 (15)
13. Fighting over small things	Fighting over small things (e.g., toys, TV remote)	5 (15)
14. Not listening	Arguing, getting angry	4 (12)
15. Not giving time	Due to parents not giving time to the child, due to coming home late from work	3 (9)
16. Due to making a lot of noise	Playing songs loudly, screaming	3 (9)
17. Discrimination	Discrimination, pride	3 (9)
18. Shortage of food/drink	Shortage of food/drink	3 (9)
Impact		
1. Bad/wrong behaviors	Not being happy, being irritable, staying angry, staying quiet, crying, breaking things, strange behavior, staying away, fighting, not eating food, not listening/obeying, learning swear words, being anxious, enmity, threatening	29 (88)
2. Not feeling interested in studies	Not able to concentrate on studies, not going to school, future being ruined	17 (52)
3. Bad effect on the mind	Running away, leaving the house, suicide, bad effect on the mind, staying sick	16 (48)
4. Feeling unsafe/unprotected	Lonely, lack of peace, sad	14 (42)
5. Feeling embarrassed	Feeling shame/dishonor, not calling friends over to the home	11 (33)
6. Thinking a lot about the fights at home	Feeling troubled, thinking about the fights at home, explaining to older people [who are fighting], fear of separating	10 (30)
7. Not feeling interested in playing/doing work	Not feeling interested in playing/doing work	10 (30)
8. Learning to fight from elders	Learning to fight from watching parents fight, talking like elders, fighting with elders	8 (24)
9. Not listening/obeying elders	Seeing them as the culprit	7 (21)
10. Using substances	Using substances, cigarettes, alcohol	7 (21)

11. Feeling sad	Not being happy, not feeling interested in work, becoming timid	7 (21)
12. Abusing (verbal)	Abusing (swearing), speaking back, yelling, threatening, making faces	5 (15)
13. Hitting	Getting together w/ friends and hitting, fighting with friends, kicks, punches	5 (15)
14. Having a bad image of one's parents	Bad image from seeing parents fight, image of parents being spoiled, not talking, living in fear, staying away	4 (12)
15. Relationships going bad	Not calling relatives (hosting), marriage prospects being spoiled	4 (12)
What people currently do		
1. Explaining	Giving advice about not fighting, explaining, elders and relatives explain, breaking up fights, removing suspicions, explaining to husband and wife	29 (88)
2. Taking help	Taking help of the police, slum president, women's groups, getting [fighters] threatened by the police [to stop fighting], calling a village assembly/jury, explaining to both parties	25 (76)
3. Mediation	Mediation	11 (33)
4. Education about morals/ethics	Love, respect, loving younger ones, no abusing	4 (12)
5. Making them admit their mistakes, resolving	Making them admit their mistakes, resolving	4 (12)
6. Leaving the house	Leaving the home and living someplace else	3 (9)
What people should do		
1. Explaining	Explaining that fighting is a bad thing, elders and relatives explain, removing suspicion	21 (64)
2. Taking help	Taking help from the police, jail, more police posts in the slum, calling village assembly/jury, taking help from the women's group	17 (52)
3. Giving good education	Giving good education, lessons about unity	8 (24)
4. Maintaining peace in the slum	Maintaining peace in the slum, making laws, explaining importance of love	6 (18)
5. Keeping people who are fighting apart	Send them to live separately if not listening	5 (15)
6. Increasing awareness/counseling	Doing a rally to stop fighting, counseling, doing plays/drama, doing home visits to explain	4 (12)
7. Self-defense training	Self-defense training	3 (9)

8. Taking child out/sending far away	Taking children out (for recreation), sending children far away	3 (9)

[a] Reported by three or more respondents.
