# Supplementary material for: A qualitative study of mental health problems among children living in New Delhi slums
Source: Transcult Psychiatry. 2024 Feb 23;61(4):533–56. doi: 10.1177/13634615231202098 (PMC11538746; doi:10.1177/13634615231202098)
Supplement: sj-pdf-4-tps-10.1177_13634615231202098 - Supplemental material for A qualitative study of mental health problems among children living in New Delhi slums [file sj-pdf-4-tps-10.1177_13634615231202098.pdf]

Supplemental Table 4. Depression and Anxiety Symptoms ($N = 33$)[a]

Cover Term	*Included Terms*	*Frequency (%)*
Signs & Symptoms		
1. Isolating/feeling lonely	Being lost in one's own world, not listening [obeying], appearing beat down, being silent/going to silent/peaceful places, appearing frightened, feeling bad, staying away from parents & society	29 (88)
2. Madness/anger	Fighting, harming others/causing them some loss, breaking things, abusing/swearing, passing sarcastic/critical remarks, mischievous behavior, yelling, torturing others, wandering, mad/demented, talking to oneself	29 (88)
3. Negative thinking/bad thoughts	Thinking one is lesser than others, perceiving something good to be bad, thoughts about running away from home, killing the person who is harassing me, suicide, taking extra pills, being jealous of others	27 (82)
4. Not eating food	Not feeling hungry/thirsty [losing appetite], not paying attention to food/drink	22 (67)
5. Irritability	Not speaking properly/being rude, talking back/not giving a straight answer, not being respectful, feeling restless	22 (67)
6. Physical weakness/pain in the mind	Falling sick, pain in hands, body, legs, and head/brain, shortage of blood, feeling dizzy	15 (45)
7. Feeling sad	Feeling strange, not laughing, forgetting the language of love, looking downcast/dispirited, sadness on face, bad mood, feeling gloomy/sad, face fallen	13 (39)
8. Taking things to heart	Anything that happens impacts [the child] heavily, taking things that happen/what people say to heart, feeling depressed due to a girl/being in love	12 (36)
9. Not engaging in any entertainment/recreation	Not having fun, not watching TV, not going out for pleasure, not going to parties	11 (33)
10. Thinking too much	Thinking about the same thing over and over again; impacting one's mind and body, thinking deeply/in-depth, thinking in one's mind, thinking day & night, thinking about why parents fight, thinking about one's friends	9 (27)
11. Being sluggish/lazy	Wasting/spoiling time, not feeling like getting up, lying down	9 (27)
12. Not feeling interested/not finding pleasure in doing things	Not interested in playing/doing work, don't have strength to think/understand	7 (21)
13. Feeling confused	Thinking while walking on the street, not doing work well	7 (21)
14. Being afraid	Living in fear, feeling afraid due to mental stress/tension	6 (18)

15. Throwing tantrums	Not listening [to what someone else is saying to do]	6 (18)
16. Not taking care of oneself	Not bathing, not taking care of one's body	6 (18)
17. Keeping things in one's heart/hiding things	Not sharing one's troubles/what's happening [or what one's feeling] with anyone	5 (15)
18. Not able to sleep	Not able to sleep	5 (15)
19. Reduced ability to think and understand	Say you'll do one thing and do another thing, hear one thing and understand something different, being forgetful	5 (15)
20. Crying	Crying	4 (12)
21. Feeling nervous/anxious	Feeling nervous/anxious, feeling suffocated, restless in the body	4 (12)
Causes		
1. Tension about studies	Depression, not getting a good education, studying for a long time, going in depth, tension about exams, failing in studies	25 (76)
2. Family tension	Tension about family members, parenting fighting, parents hitting children, fighting among relatives	24 (73)
3. Basic needs not being met	Lack of money, not getting clothes, not getting favorite food, not being taken care of	21 (64)
4. Fighting	Fighting among friends, fighting among parents due to friends, fighting among children	16 (48)
5. Not speaking properly/being irritable	Being irritable, not talking to parents	14 (42)
6. Taunting child/passing sarcastic remarks	Taunting child about their appearance, elders disrespect/disgrace the child, taunting child about living in a slum, wrongfully being blamed by the family, not speaking properly [to the child]	12 (36)
7. Making comparisons to other children	Comparing oneself to others, comparing oneself, thinking one is weak	11 (33)
8. Alcohol & drug use	Parents using substances, children doing substances, doing substances after watching other people do it, such as alcohol, marijuana, smoking cigarettes	11 (33)
9. Harassment	Depression due to being harassed, [children] are tortured, taunting/passing sarcastic remarks [towards harassed child], [harassed child] threatening to die, threatening to tell the police, telling parents about harassment, ragging/hazing, wrongfully blaming the child	11 (33)
10. Tension about the environment	Seeing the neighborhood/environment, seeing spoiled [bad] people	10 (30)

11. Not getting love	Not getting love from parents, not being spoken to, not getting love & care from a young age, [experiencing] injustice	9 (27)
12. Tension about goals/career	Tension about goals (career)	9 (27)
13. Being married at a young age	Being married at a young age, being deceived/cheated in love, being married due to failing [in school], talking about marriage at a young age, being forced to marry, have sex, not being married to the person of your choice	8 (24)
14. Sexual harassment	Teachers raping child, being raped at a young age, becoming pregnant at a young age	6 (18)
15. Falling into bad company	Falling into bad company, being deceived while pursuing girls, bad/narrow thoughts	5 (15)
16. Another person's death	Death of family members	5 (15)
17. Not telling anyone what happened	Not telling anyone what's on one's mind or what happening, for e.g., about rape; fear of parents being dishonored or fear of there being a fight	4 (12)
18. Pressure at home/oppression	Older children oppress younger children, pressure to do household work, having a lot of responsibility from a young age, pressure to study	4 (12)
19. Not engaging in entertaining activities	Not playing, not watching TV, not going to family parties	4 (12)
20. Discrimination between boys & girls	Giving preference to boys when there is a shortage of food & drink, making boys study more but not girls, not meeting [girl's] needs, discrimination between boys & girls	3 (9)
21. Mistrust/lack of understanding from family	Family not believing what the child is saying, family not understanding, giving the wrong answer	3 (9)
Impact		
1. Negative impact on child’s studies	Being away from education, not studying, not feeling interested in studying, not being able to concentrate, impact on tuition [i.e., extra after-school classes], tension about exams	26 (79)
2. Mental tension/stress	Tension between brothers and sisters, domestic violence, tension visible on the face, tension due to being harassed, not doing work due to tension	24 (73)
3. Negative impact on the child’s family	Family is sad, worried, thinking badly about the child (blaming the child), worrying about getting treatment, thinking/wondering about what things are lacking in the child's life, feeling pressure to send the child far away, worrying that the child might run away from home, parents hurting	21 (64)

4.	Negative impact on the community	Feeling scared (that child will harm them/cause loss), staying away/far [from the child], thinking the child is bad, worrying that the child will have/has a bad influence on other kids, ignoring what the child says, thinking about if the child throws stones or gets into the house or breaks things in the house [worrying about these things happening]	19 (58)
5.	Bad behaviors	Running away from home, stealing	17 (52)
6.	Using substances	Smelling eraser fluid, falling into bad company, using substances to get rid of tension, not having control over one's body, using substances after seeing others use them, smoking cigarettes, not listening to people at home due to using substances	14 (42)
7.	Committing suicide	Hanging, eating poison, or committing suicide [through other means] due to studies, not getting love, harassment and rape	11 (33)
8.	Not going to school/college	Not going to school, stop studying [drop out]	8 (24)
9.	Hurting oneself	Taking the depression out [by cutting], cutting one's hand with a blade, hitting one's head on the wall	7 (21)
10.	Career being ruined	Dreams not being fulfilled, worrying about career	7 (21)
11.	Threaten to commit suicide	Threaten to commit suicide	3 (9)
12.	Ruining one's life	Changing one's way of living, not knowing the difference between right and wrong, making one's own society to live in	3 (9)
What people currently do			
1.	Parents explaining child's situation	Parents explaining child's problems to the school teacher	16 (48)
2.	Doing fun/pleasurable activities with the child	Taking the child for an outing [take outside, for pleasure, roaming around], playing, listening to music, telling the child stories	16 (48)
3.	Giving the child more love and time	Taking the child out of their tension, giving the child time/spending more time with the child	14 (42)
4.	Taking care of child's needs	Parents take care of the child's needs (clothes, books, good friends), buying the child games, dolls, their favorite thing	13 (39)
5.	Creating a nice environment	Living in harmony, not troubling child, not asking child too many questions	13 (39)
6.	Keeping child away from stress	Keeping child away from depression, helping the child have good friends, not getting angry	12 (36)
7.	Giving advice & getting treatment	Giving advice about treatment, getting treatment (sending to the doctor), listening to [child's] problems	10 (30)

8.	Sending child far away	Sending the child far away (like the village), making the child meet relatives/neighbors	8 (24)
9.	Sharing thoughts	Sharing everything (all things on one's mind) with the child, being the child's friend	5 (15)
10.	Counseling at the NGO	Getting counseling at Asha/NGO, others counseling/explaining to the child	4 (12)
11.	Providing school/career support	Sending child to a good place for tuition [i.e., extra after-school classes], talking about their career, sending child to school	4 (12)
What people should do			
1.	Providing entertainment/not letting the child be alone	Doing entertaining/fun activities with children (playing games, TV, films, going for an outing, watching sports, dance, music, exercise, walking), taking the child to historic places (e.g., qutab minar, jantar-mantar, mughal gardens), should try and remove the child's loneliness	25 (76)
2.	Parents doing good behaviors with children	Speaking nicely/cheerfully to children, keeping a good environment at home, raising them with love, giving them attention/taking care, spending time with children, trying to understand what's in their heart as much as possible, encouraging them, not abusing/using swear words	24 (73)
3.	Parents should fulfill child's needs	Environment, basic necessities, friends, food & drink, sending them to school)	14 (42)
4.	Creating a good environment/keeping good company	Doing things with good children, staying in good company	10 (30)
5.	Parents doing good behaviors in the slum	Parents maintaining friendships/good relations in the slum, not fighting, understanding	8 (24)
6.	Getting treatment from a doctor	Sending [child] to the doctor, showing the child to the doctor [getting treatment]	7 (21)
7.	Child sharing their thoughtsa	[Child should] share openly, even the smallest of things, have child talk to good friends who they can tell their problems to	6 (18)
8.	Staying away from things that give stress	Not doing things that cause stress, staying away from fighting and using substances	6 (18)
9.	Getting love and support from friends	Getting love from friends, should explain [child's problems] to other children so that the child doesn't have damage	5 (15)
10.	Not putting pressure on child about studies and their goals	Not putting pressure on child about studies and their goals	4 (12)
11.	Counseling/going to the NGO	Should take help of the NGO, get counseling	4 (12)

12. Have meetings to talk about children's problems	Talk about the problems children face, understanding [child's problems]	3 (9)
13. Motivate the child	Give them training/skills to face pressure/skills	3 (9)

[a] Reported by three or more respondents.
